# Hybrid nanodiamond quantum sensors enabled by volume phase transitions of hydrogels

**DOI:** 10.1038/s41467-018-05673-9

**Published:** 2018-08-09

**Authors:** Ting Zhang, Gang-Qin Liu, Weng-Hang Leong, Chu-Feng Liu, Man-Hin Kwok, To Ngai, Ren-Bao Liu, Quan Li

**Affiliations:** 1 Department of Physics, The Chinese University of Hong Kong, Shatin, New Territories, Hong Kong China; 2 Department of Chemistry, The Chinese University of Hong Kong, Shatin, New Territories, Hong Kong China; 3 Shenzhen Research Institute, The Chinese University of Hong Kong, Shenzhen, 518100 China; 4 Centre for Quantum Coherence, The Chinese University of Hong Kong, Shatin, New Territories, Hong Kong China

## Abstract

Diamond nitrogen-vacancy (NV) center-based magnetometry provides a unique opportunity for quantum bio-sensing. However, NV centers are not sensitive to parameters such as temperature and pressure, and immune to many biochemical parameters such as pH and non-magnetic biomolecules. Here, we propose a scheme that can potentially enable the measurement of various biochemical parameters using diamond quantum sensing, by employing stimulus-responsive hydrogels as a spacing transducer in-between a nanodiamond (ND, with NV centers) and magnetic nanoparticles (MNPs). The volume phase transition of hydrogel upon stimulation leads to sharp variation in the separation distance between the MNPs and the ND. This in turn changes the magnetic field that the NV centers can detect sensitively. We construct a temperature sensor under this hybrid scheme and show the proof-of-the-principle demonstration of reversible temperature sensing. Applications in the detection of other bio-relevant parameters are envisioned if appropriate types of hydrogels can be engineered.

## Abstract (web summary)

Nitrogen-vacancy (NV) centers in diamonds are used for quantum sensing but NV centers are not sensitive to parameters such as temperature, pressure and biomolecules. Here the authors propose a scheme based on a magnetic nanoparticle docked responsive hydrogel which acts as a transducer between the particles and the diamond.

## Introduction

Nitrogen-vacancy (NV) centers in diamond are promising quantum bio-sensors due to their high photo-stability and long spin coherence times under ambient conditions^1,2^. In addition, the available small size of the diamond nanoparticles (NPs) enables spatially resolved sensing applications, making it possible to study cellular machinery at the subcellular level. However, while the spin states of NV centers respond most sensitively to magnetic fields^3,4^, they do much less sensitively to changes in parameters such as temperature and pressure^5–7^, and not at all to a broad range of parameters including pH, sugar, and non-magnetic ions and biomolecules. To exploit the advantages of diamond quantum sensing for studying the broad range of parameters relevant to cellular machinery, it is highly desirable to convert variations of such parameters to magnetic signals by a transducer. The transducer should be designed to respond to specific stimuli and produce a signal that enables the sensitive sensing of NV centers, such as a piezomagnetic layer above diamond NV centers that can convert the change in the stress to a magnetic field variation^8^.

In this work, we present the proof-of-the-principle demonstration of a transducer-based reversible quantum sensor^9^. The key idea is to employ a stimulus-responsive hydrogel as the transducer. The hydrogel is grafted around an ND to form a shell, and then a few MNPs are docked on the hydrogel shell. The hydrogel undergoes a volume phase transition upon the change of a specific parameter in the range of interest^10^. The volume phase transition of the hydrogel shell and hence the sharp distance variation between the ND and the MNPs induces a large change of the magnetic field that can be sensed by the NV centers via optically detected magnetic resonance (ODMR)^11,12^. We take the temperature-responsive poly(*N*-isopropylacrylamide) (pNIPAM) hydrogel as the model system^13^, and construct hybrid sensors of ND@pNIPAM–MNPs. We carry out temperature sensing with a single hybrid sensor and demonstrate a modestly enhanced sensitivity and good reversibility of the nanosensor. The hybrid scheme can be potentially extended to the detection of a broad range of biochemical parameters, when the hydrogel is chemically engineered to be responsive to the parameters (glucose, pH, enzyme, etc.)^14–16^.

## Results

### Proposal of hydrogels as transducers for hybrid ND sensors

**Fig. 1:**
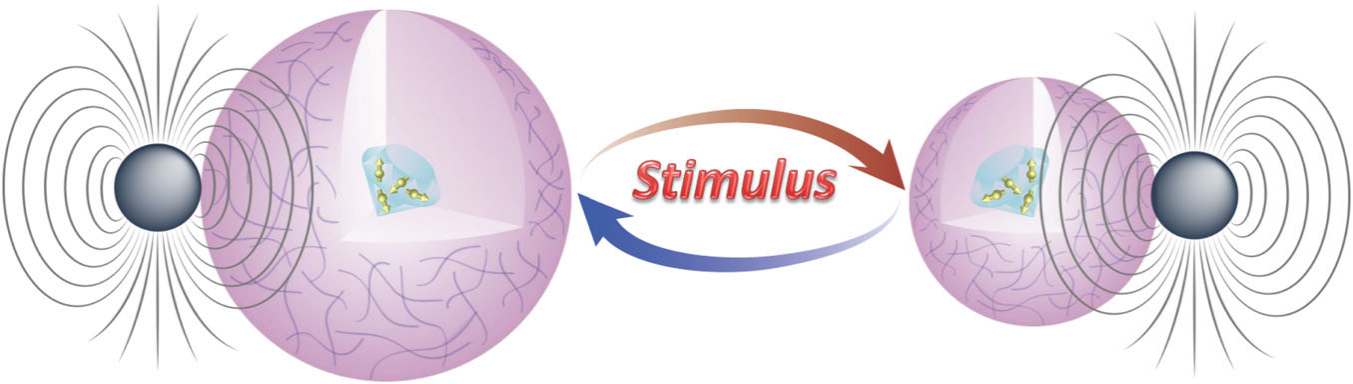
Schematic of an ND@hydrogel–MNP hybrid sensor

The hybrid nanosensor is designed to have an ND as the core, a shell of hydrogel, and surface-docked MNPs. The schematic is shown in Fig. 1. The ND contains an ensemble of NV centers. The NV center spin resonances are split by the magnetic field from the MNPs (if the field is uniform) or broadened by the field (due to the large gradient, which is common at the nanoscale). The hydrogel can be chemically engineered to be responsive to a designated parameter (pH, glucose concentration, enzyme concentration, etc.). When the parameter varies near the critical value, the hydrogel shell will undergo a volume phase transition. The volume phase transition induces a sharp change of the distance between the ND and the MNPs^10^, and in turn a sharp change in the NV center spin resonance splitting and/or broadening, which can be readily measured by ODMR^11,12^. The ODMR spectrum therefore constitutes a sensitive measurement of the parameter near the critical point.

For a specific demonstration, pNIPAM is chosen as a model system for the transducer. pNIPAM is a well-known thermal sensitive polymer with lower critical solution temperature (LCST) at about 32 °C^13^. The LCST can be varied by modifying the cross-linking density of the hydrogel network^17^. Below its LCST, pNIPAM is in its swollen state (fully hydrated). When temperature increases close to its LCST, collapse of pNIPAM takes place, and hence a volume shrinkage^18^. The collapse results in a decrease in the separation distance between the ND and the MNPs, and thus an increase in the magnetic field on the NV centers. The phase transition of pNIPAM with temperature is reversible^19^, making it promising for constructing reversible hybrid quantum sensors.

The NDs have an average size of ~200 nm (polydispersity index, PDI ~0.25; standard deviation, *σ* ~70 nm) and contain ~500 NV centers per ND. Such NDs have large photon count rates (hence good signal-to-noise ratio). For the MNPs, single domain ferromagnetic NPs are preferred for their large and stable magnetic moments in the absence of an external magnetic field. In the present work, Ni NPs are chosen for such a purpose. Bulk Ni is ferromagnetic at room temperature with saturation magnetization of 484 kA m^−1^ ^20^, and the largest single domain size is estimated as with a diameter of ~53 nm^21^. The working separation distance for the hybrid sensor is determined by the magnetic moment of the MNPs and the size of the ND. The magnetic field from the MNPs causes the splitting of the NV center spin resonances as well as broadening due to the field gradient within the ND (especially when the MNPs are close to the ND). This imposes the requirement on the thickness of the hydrogel—it needs to be sufficiently small for NV centers to detect the induced magnetic field gradient as well as field variation upon external stimulation (for the magnitude of the magnetic field from the MNPs, see Supplementary Fig. 1). On the other hand, it should be large enough to reduce the field gradient and hence the ODMR spectrum broadening for better sensitivity. For an ND with size ~200 nm and Ni MNPs ~50 nm, as employed in the present work, the optimal thickness for the hydrogel shell should be ~100–200 nm. A more thorough discussion on the effect of hydrogel shell thickness is given in later sections and Supporting Information (Supplementary Fig. 16).

### Preparation of ND@pNIPAM–Ni hybrid sensors

**Fig. 2:**
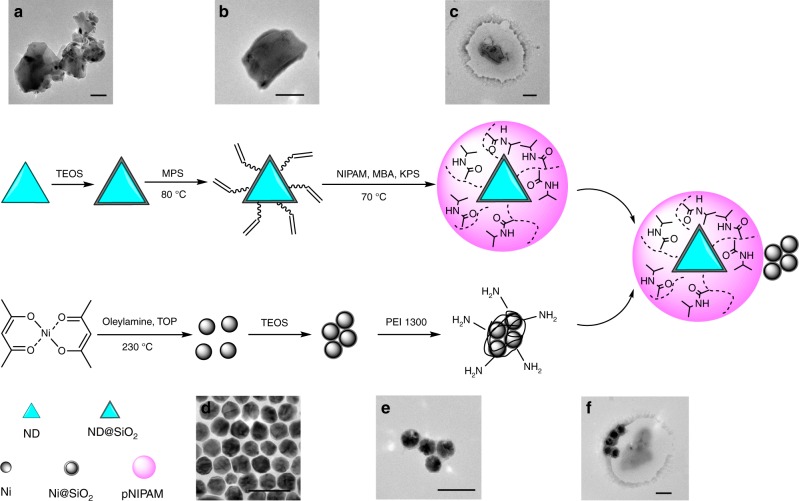
Synthetic scheme of the ND@pNIPAM–Ni hybrid sensor and the TEM characterizations. Typical TEM images of **a** bare NDs, **b** an ND@SiO_2_-MPS particle, **c** an ND@pNIPAM particle, **d** bare Ni NPs, **e** an Ni@SiO_2_-NH_2_ cluster, and **f** an ND@pNIPAM–Ni hybrid particle. Scale bar is 100 nm

The synthesis scheme of the hybrid sensor is illustrated in Fig. 2. Details of the procedure can be found in the Methods section. ND (500 NV) with an average diameter of ~200 nm is employed in the present work. Aggregation of the bare NDs is severe (Fig. 2a), but can be much improved (Fig. 2b) when the surfaces of the NDs are modified with SiO_2_ (Supplementary Fig. 2). After further modification with [3-(methacryloyloxy)propyl]trimethoxysilane (MPS), the presence of carbon–carbon double bonds (Supplementary Fig. 3) on the ND@SiO_2_ surface allows grafting of pNIPAM to form a hydrogel shell through free radical precipitation polymerization^22^. Transmission electron microscope (TEM) (Fig. 2c) shows the pNIPAM shell with a thickness of about 100–200 nm (in dried state on carbon film). The ND@pNIPAM core–shell structure has an average size of ~540 nm (PDI ~0.11, *σ* ~140 nm) with little aggregation observed (Supplementary Fig. 4) at room temperature. Successful grafting of pNIPAM on NDs is also evidenced by confocal microscopy. The high ratio of overlap between the fluorescence signals of pNIPAM and NDs suggests their effective coupling (Supplementary Fig. 5). The grafted hydrogel shells are stable for at least 5 months. The surfaces of the ND@pNIPAM NPs are negatively charged with zeta potential about −20 mV (*σ* ~5 mV), as suggested by the zeta potential measurement (Supplementary Fig. 9a). The as-synthesized Ni NPs have the face-centered cubic (FCC) structure (Supplementary Fig. 6). The average size of the Ni NPs is ~50 nm (Fig. 2d). Vibrating sample magnetometer (VSM) measurement of the as-synthesized NPs shows a saturated magnetization of about 28 emu g^−1^ (Supplementary Fig. 7). A surface SiO_2_ coating with amine modification has been grown on the as-synthesized Ni NPs, which subsequently forms clusters of 2–6 Ni NPs. The morphologies of the Ni clusters are characterized by TEM (a typical image shown in Fig. 2e). The surfaces of these Ni clusters are positively charged with a zeta potential of ~43 mV (*σ* ~9 mV) (Supplementary Fig. 9b). Dynamic light scattering (DLS) measurement (Supplementary Fig. 8) shows that the size of the modified Ni clusters is about 150 nm (PDI ~0.20, *σ* ~50 nm), which is consistent with the TEM observation. The ND@pNIPAM–Ni hybrid sensor (Fig. 2f, for more images, see Supplementary Fig. 10) is lastly constructed by electrostatic interaction between the negatively charged ND@pNIPAM and positively charged Ni clusters.

**Fig. 3:**
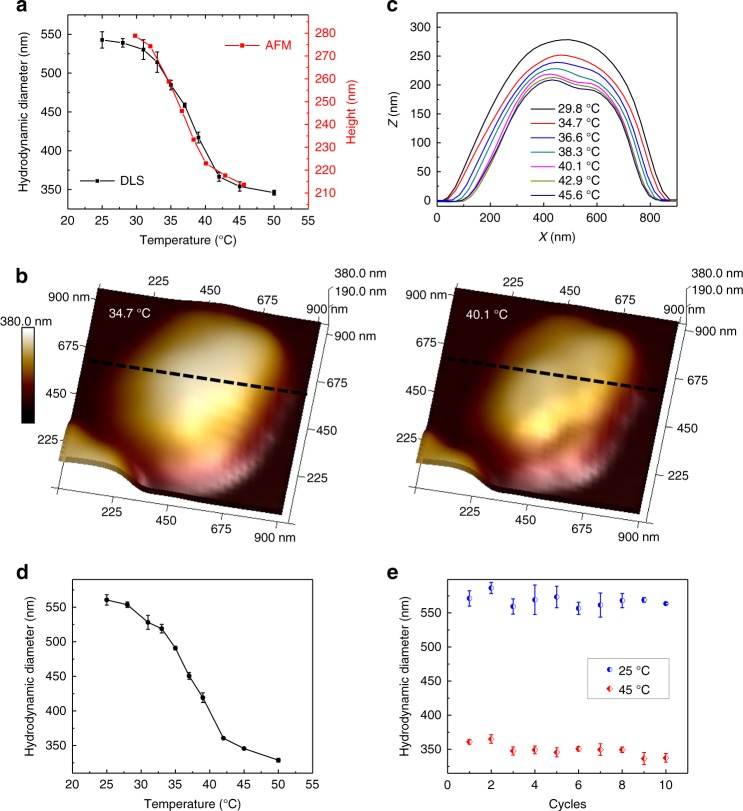
Collapse/swelling test of ND@pNIPAM and ND@pNIPAM–Ni. **a** Temperature-dependent hydrodynamic diameters of ND@pNIPAM NPs measured by DLS (black) and temperature-dependent height of an ND@pNIPAM NP on a Si substrate measured by AFM (red). **b** Typical topographies of an ND@pNIPAM NP measured by AFM below (34.7 °C) and above (40.1 °C) the LCST, and **c** the corresponding height profiles of the ND@pNIPAM NP at different temperatures on Si substrate measured by AFM, taken as positions indicated by the black dashed lines in **b**. **d** Temperature-dependent hydrodynamic diameters of ND@pNIPAM–Ni hybrid sensors measured by DLS. **e** The reversibility test of ND@pNIPAM–Ni hybrid sensors at 25 °C (blue) and 45 °C (red). The vertical error bars of DLS results in **a**, **d**, **e** are the standard errors of median for three measurements

The thermo-response of an ensemble of ND@pNIPAM is first characterized by DLS, as shown in Fig. 3a (black line). The average hydrodynamic diameter of ND@pNIPAM changes from ~540 nm at 25 °C to ~350 nm (PDI ~0.06, *σ* ~80 nm) at 50 °C. By subtracting the average ND core size (including the SiO_2_ coating) of ~220 nm (PDI ~0.12, *σ* ~80 nm), the thickness change in the pNIPAM shells is estimated to be from ~160 nm at 25 °C to ~65 nm at 50 °C, with a diameter swelling ratio of ~2.5. The fact that the size distributions before and after the pNIPAM coating are similar suggests the relatively uniform distribution of the shell thickness. The thickness of the pNIPAM layer is within the optimal range. The LCST of ND@pNIPAM is estimated as ~37 °C from the DLS data.

The swell–collapse behavior of individual ND@pNIPAM NPs on a substrate is further investigated using atomic force microscopy (AFM). Figure 3b shows typical topographies of an ND@pNIPAM particle on a Si substrate below (34.7 °C) and above (40.1 °C) the LCST, disclosing the morphologies of the ND@pNIPAM particle at the swollen and collapsed states, respectively. The corresponding height profiles (taken from positions marked in Fig. 3b) are plotted in Fig. 3c, revealing the general trend of collapsing with temperature increase. As shown in Fig. 3a, the temperature-dependent AFM and DLS results agree well with each other. The smaller height value and the larger lateral size of the ND@pNIPAM measured by AFM (as compared with the hydrodynamic diameter of the same particles measured by DLS) could result from the size variation of a specific ND core (height range from ~65 to ~175 nm measured by AFM, and diameter range from ~50 to ~400 nm measured by TEM, based on silica-coated NDs), as well as wetting of the hydrogel on the Si substrate. Nevertheless, neither the size difference in NDs nor the wetting of hydrogel on the Si substrate affects the temperature-stimulated swelling/collapse of the ND@pNIPAM NPs (Fig. 3c).

The temperature-stimulated swollen–collapse transition of the ND@pNIPAM–Ni hybrid sensors is investigated by DLS (Fig. 3d). The hybrid sensors present hydrodynamic diameter variation as a function of temperature consistent with the DLS measurement of ensemble ND@pNIPAM NPs, and the LCST of the hybrid sensor is identical to that of the ND@pNIPAM NPs. The temperature response of the ND@pNIPAM–Ni hybrid sensors is shown to be reversible by DLS measurement in repeated heating–cooling cycles between 25 and 45 °C (Fig. 3e).

### Temperature sensing

**Fig. 4:**
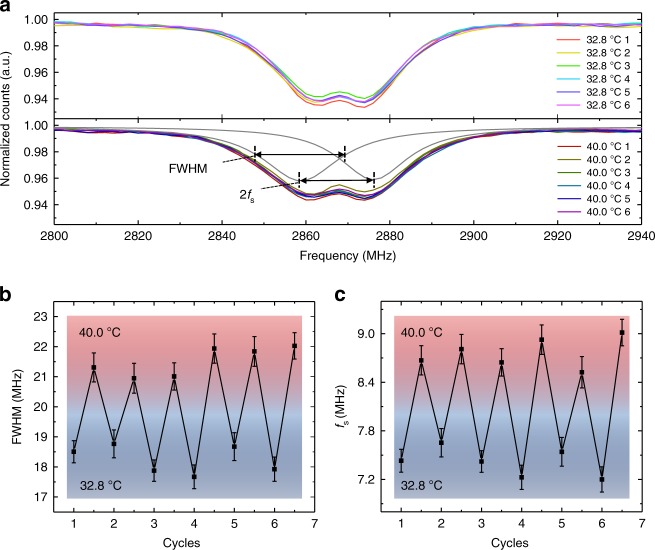
Reversibility test of the hybrid sensor for temperature sensing. **a** Typical ODMR spectra of a single hybrid sensor taken below LCST (32.8 °C, swollen state, upper pane) and above LCST (40.0 °C, collapsed state, lower pane). The measurements at high and low temperatures are carried out sequentially and repeated for six cycles. The two-peak Lorentz fitting is shown as gray lines. **b** The average FWHM of the two fitted peaks, and **c** the peak shift *f*_s_ measured in six cycles of alternating low and high temperatures. The vertical error bars in **b**, **c** are the fitting errors

The temperature responses of the hybrid sensors are characterized by ODMR measurements on a home-built confocal microscope (details of the setup and method shown in Supplementary Fig. 11). The sample temperature is controlled by a thermoelectric cooler (TEC) and calibrated by the resonant frequencies of the sensor itself (details in Supplementary Note 5). Spin resonance spectra of single ND@pNIPAM–Ni nanosensor are firstly measured at a temperature below LCST (32.8 °C) and then above LCST (40.0 °C), as shown in Fig. 4a. At both temperatures, a two-peak feature with large broadening is observed, consistent with the fact that the magnetic field from the Ni clusters has a gradient among the spatially distributed NV centers in the ND. Lorentz double-peak fitting of the ODMR spectra (gray lines in Fig. 4a) gives the peak shift *f*_s_ (defined as the difference between the frequency of a split peak and the zero field frequency), and the full width at half maximum (FWHM) of each fitted peak (details of the fitting method can be found in Supplementary Note 4). At 32.8 °C, relatively narrow peaks (FWHM from 17.7 to 18.8±0.5 MHz (fitting error)) are obtained and the peak shifts are in the range of 7.2 to 7.6±0.2 MHz (fitting error) (Fig. 4b, c). In comparison, broader peaks (FWHM from 20.9 to 22.0±0.5 MHz (fitting error)) are observed at 40.0 °C, and large peak shift is observed (from 8.5 to 9.0±0.2 MHz (fitting error)) (Fig. 4b, c). The increases of the peak broadening and splitting with temperature rising suggest the corresponding increases of magnetic field and field gradient, which result from the hydrogel collapse above the LCST and consequently the reduction of separation between the Ni NPs and the ND. Both the peak shift and width present good repeatability as the temperature is alternatively set at the low and high values, suggesting the reversibility of the hybrid sensor (Fig. 4b, c).

**Fig. 5:**
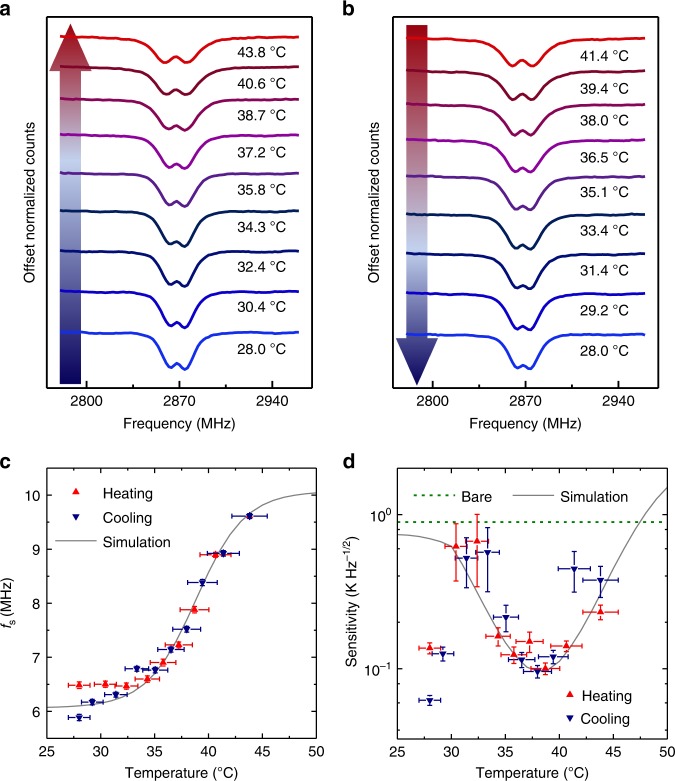
Temperature sensitivity of the hybrid sensor. **a**, **b** ODMR spectra of an ND@pNIPAM–Ni hybrid sensor at different temperatures during the heating and cooling processes, respectively. **c** Temperature-dependent peak shift *f*_s_, and **d** the estimated sensitivity at different temperatures in the heating (red) and cooling (navy) cycles, in comparison with numerical simulation (gray). Olive dash line in **d** indicates the sensitivity of the bare ND temperature sensor in the absence of MNPs. The horizontal error bars in **c**, **d** are the fitting errors of the temperature calibrations. The vertical error bars in **c** are the fitting errors, and in **d** are the shot-noise errors from the ODMR spectra

To assess the temperature sensitivity of the hybrid sensor, the ODMR spectra are measured for both heating and cooling processes at a series of fine-tuned temperatures (Fig. 5a, b). Figure 5a, b shows a few representative ODMR spectra, which appear similar to those in Fig. 4. Both peak frequency and width changes are detected with temperature increase/decrease. Figure 5c plots the fitted temperature-dependent peak shifts (obtained by fitting of the ODMR spectra). The good match between the measurements in the heating and cooling cycles suggest reversibility of the hybrid sensor. An LCST transition temperature of ~37 °C is estimated from the temperature-dependent peak shift (Fig. 5b), which is typical among all sensors tested and consistent with DLS measurement. The temperature susceptibility of the peak shift is estimated to be ~0.5 MHz K^−1^ at the LCST.

The shot-noise-limited sensitivity *η*_T_ of the hybrid thermometer is estimated (Fig. 5d) by the variation of photon counts with temperature change for the microwave frequency set at the optimal value (details shown in the Methods section and Supplementary Note 7). The fluorescent photon count rate in the experiment is obtained as *L* ~8.0×10^6^ s^−1^. Optimal sensitivity of the hybrid sensor is estimated to be 96±9 mK Hz^−1/2^ (shot-noise error from the ODMR spectrum) around the LCST, nearly one order of magnitude improved from the sensitivity of a bare ND, 900 mK Hz^−1/2^ (olive dashed line in Fig. 5d), which is estimated with the same photon count rate as the hybrid sensor but with the zero-field splitting shift (d*D*(*T*)/d*T* = −74 kHz K^−1^) and broadening (13.3 MHz) of the ND in the hybrid sensor at low temperature (28 °C, at which the MNPs have negligible effects and therefore the broadening is close to the intrinsic value of the bare ND) (details shown in Supplementary Note 7). In addition, the temperature sensitivity of the hybrid sensor at the optimal temperature has one order of magnitude improvement from that of bare NDs reported earlier (~1 K Hz^−1/2^)^23^. We also conduct ODMR measurements of the hybrid sensor in fetal bovine serum (FBS), and the sensitivity in FBS is similar to that measured in water (details shown in Supplementary Note 8).

We carry out simulation of the ODMR spectra of a hybrid sensor for various pNIPAM shell thickness. The simulation employs a simplified model, including an ND of ~200 nm, a pNIPAM shell, and a typical MNP cluster of four Ni MNPs (each with 50 nm diameter) docked on the pNIPAM shell (Supplementary Fig. 16a). Using the typical temperature-dependent thickness of pNIPAM measured by DLS (Fig. 3a), we obtain the temperature-dependent peak shift from the simulated ODMR spectra (Supplementary Note 9) and therefore the temperature sensitivity. The simulation results agree well with the experimental data (Fig. 5c, d). The best sensitivity in the simulation (96 mK Hz^−1/2^) is close to the experimental value. The sensitivity is also estimated for different hydrogel thickness and different effective moment of Ni clusters (as compared with the moment *M*_0_ of single Ni NPs). TEM shows that a typical hybrid sensor consists of a cluster of 2–6 Ni NPs. The net moment of such a Ni cluster is estimated to be similar to that of a single Ni NP (see Supplementary Note 9). The simulation suggests that the optimal sensitivity is mostly 60–200 mK Hz^−1/^^2^ for hydrogel thickness from 80 to 300 nm and effective moment from 0.1 to 2 *M*_0_.

## Discussion

The sensitivity can be improved by increasing the photon count rate and the temperature susceptibility of the spin resonance frequencies and broadening. The temperature susceptibility is determined by the magnetic field strength and gradient and the collapse ratio of the hydrogel. A straightforward way to increase the temperature susceptibility is to increase the effective magnetic moments of the MNPs, by choosing larger MNPs and materials with stronger magnetization. For example, Co MNPs have larger magnetization at room temperature (1422 kA m^−1^ in comparison with 484 kA m^−1^ of Ni) and also larger single-domain size (~96 nm in comparison with ~53 nm of Ni MNPs)^21^. Another way is to optimize the thickness of the hydrogel shell. Smaller thickness would on the one hand enhance the magnetic field strength in the ND (and hence temperature susceptibility), but on the other hand increase the broadening due to field gradient and reduce the change of the distance between the ND and the MNPs (with the collapse ratio assumed constant). Using a relatively small ND (without reducing the photon count rate) can help alleviate the broadening effect. In the simulation presented in Supplementary Fig. 17a, we show that an optimal sensitivity ~31 mK Hz^−1/2^ is achievable by using a 50 nm ND containing 100 NV centers^24^ (preparation of samples of such quality, however, is still challenging), and a 90 nm Co MNP (which has a total magnetic moment about 17 times that of a Ni MNP of 50 nm diameter), using the same photon count rate (8×10^6^ s^−1^), ODMR contrast (7.5%), and hydrogel collapse ratio (0.84) as realized in the experiment. In addition, if an ND containing a single NV center is used, the optimal sensitivity can be improved to ~0.3 mK Hz^−1/2^, since the broadening effect due to the field gradient is absent (see Supplementary Note 9). However, using single-NV NDs would require higher laser power (>100 μW μm^−2^ for counts ~10^5^ s^−1^), which would introduce photo-toxicity in bio-applications.

In summary, we propose and construct an ND@hydrogel–MNP hybrid quantum sensor using stimulus-responsive hydrogel to transduce the variation of a parameter to the change of the distance between the ND and the MNPs and hence the magnetic field from the MNPs on the NV centers in the ND. The proof-of-the-principle demonstration of such a hybrid sensor is achieved by choosing pNIPAM as the model hydrogel for temperature sensing. The sensitivity is enhanced near the volume phase transition temperature of the hydrogel (~96 mK Hz^−1/2^) by nearly one order of magnitude from the sensitivity of a bare ND (~900 mK Hz^−1/2^) under the otherwise same conditions. The hybrid sensor presents remarkable reversibility and is stable for more than 1 week. Potentially it is possible to engineer hydrogels to be responsive to other parameters^14–16^ and therefore to generalize the hybrid scheme for applications in sensing other physiological parameters, such as pH, sugar, enzyme, and other types of non-magnetic biomolecules.

## Methods

### Reagents

Type Ib NDs (500 NV per particle, diameter ~90 nm as indicated by the sample label and *Z*-average diameter ~200 nm from DLS measurement, amphoteric surface groups, 1 mg ml^−1^ in DI H_2_O) were purchased from Adámas Nanotechnologies. Potassium persulfate (KPS, ≥99.0%), methylenebis(acrylamide) (MBA, 99%), fluorescein sodium salt (FSS), and potassium bromide (KBr, ≥99%, FTIR grade) were all purchased from Sigma-Aldrich without further purification. MPS (98%), *N*-isopropylacrylamide (NIPAM, 97%), tetraethyl orthosilicate (TEOS, ≥99.0%), tungstophosphoric acid (TPA, 99.995%), Igepal CO-520 (average *M*_n_ = 441 g mol^−1^) and polyethylenimine (PEI, 50 wt% solution in water, *M*_w_ = 1300 g mol^−1^) were all purchased from Aldrich without further purification. Ammonia solution (NH_3_H_2_O, 28–30%) was purchased from Merck. Nickel acetylacetonate (Ni(acac)_2_, 95%), oleylamine (OA, 80–90%) and trioctylphosphine (TOP, 90%) were all purchased from Aladdin without further purification. FBS was purchased from Thermo Fisher (Gibco) without further treatment.

### Modification of ND with MPS

Bare NDs were firstly coated with SiO_2_ through a modified Stöber method, briefly described below: 1 mg NDs were added to a mixture with 3.75 ml ethanol, 1.15 ml H_2_O, and 75 μl NH_3_H_2_O under sonication, followed by the slow addition of 15 μl TEOS; the solution was sonicated for 1 h at the power of 450 W, and then purified by three cycles of centrifugation (11,000 rpm, 5 min). The as-synthesized ND@SiO_2_ particles were re-dispersed in 20 ml isopropanol. After the addition of 400 μl MPS, the mixture was heated to 80 °C and then kept at the temperature for 2 h under reflux. The resultant MPS-modified NDs were purified by three cycles of centrifugation (11,000 rpm, 5 min), and finally re-dispersed in H_2_O for further use. FTIR measurements were carried out to confirm the MPS modification: In a typical procedure, 0.5 mg sample particles (ND@SiO_2_ or ND@SiO_2_–MPS) were mixed with 100 mg KBr; the mixtures were finely pulverized, and then loaded into a pellet-forming die, with an applied force of approximately 7 tons for about 3 min to form transparent pellets; sample of pure MPS was prepared by dropping 1 μl MPS solution on the surface of a blank KBr pellet; after drying, the pellet was then re-pulverized and re-pressed; the prepared pellets (with MPS/ND@SiO_2_/ND@SiO_2_–MPS) were loaded into the FTIR chamber one by one. Spectra of the pellets from 4000 to 400 cm^−1^ were collected.

### Grafting of pNIPAM on ND particles

ND@pNIPAM were synthesized by grafting pNIPAM to the MPS-modified NDs through free radical precipitation polymerization: Firstly, 100 mg NIPAM and 15 mg MBA (11 mol% relative to NIPAM) were dissolved in 35 ml H_2_O; after transferred to a 100 ml three-neck round bottom flask immersed in an oil bath, the mixture was heated to 70 °C and deoxygenized by continuous N_2_ flow for 1 h; then 1 mg ND–MPS dispersed in 5 ml H_2_O were injected to the solution; after deoxygenation for another 5 min, 12.5 mg KPS dissolved in 1 ml H_2_O was slowly injected into the flask to initiate the polymerization; the polymerization process lasted for 4 h; then five cycles of centrifugation (11,000 rpm, 5 min) were performed to purify the product; the as-synthesized ND@pNIPAM particles were re-dispersed in H_2_O for further use. For TEM characterization, firstly 10 μl of the sample solution was taken out and dried on the carbon film on copper grids. Then staining process was performed by dropping 10 μl of 2% neutralized TPA on above the carbon film for 10 min. Fluorescence measurement of ND@pNIPAM was performed on a confocal microscope. 20 μl FSS (0.3 mg ml^−1^) was added to 200 μl ND@pNIPAM solution (10 μg ml^−1^) to label the pNIPAM shell. The labeled solution was dropped to a confocal dish and then 20 min were allowed for the settling down of the particles. Fluorescence signals of FSS (Ex. 488 nm, Em. 500–545 nm) and NDs (Ex. 561 nm, Em. 570–640 nm) were collected.

### Synthesis of Ni NPs

Ni NPs were synthesized by high-temperature organometallic decomposition^25^: Firstly, 2 g Ni(acac)_2_, 20.8 g OA, and 0.29 g TOP were added to a four-neck round bottom flask; the mixture was heated to 120 °C at the rate of 5 °C per min under N_2_ atmosphere, and kept at the temperature for 30 min without reflux to deoxidize and dehydrate the reactant; then it was heated to 230 °C at the rate of 3 °C per min; the reaction lasted for 2 h under mechanical stirring (700 rpm); the product was firstly precipitated with 50 ml acetone, and then washed with ethanol for three times by centrifugation (11,000 rpm, 5 min); finally, the as-synthesized Ni NPs were re-dispersed in cyclohexane to yield a concentration of 10 mg ml^−1^ for further use. Morphologies of the Ni NPs were characterized by TEM. Structure and crystallinity of the particles were measured by X-ray diffraction (XRD). About 10 mg dried particles were added to the sample holder. XRD measurement was performed with 40 kV beam voltage and 40 mA beam current. The spectrum was collected from 30° to 80° (2*θ*) with steps of 0.02° and duration time of 10 min. Magnetization of the Ni NPs was detected by VSM. About 10 mg dried particles were wrapped with preservative film and then inserted to a polypropylene tube. The VSM measurement was performed at room temperature with an applied magnetic field from −10,000 to 10,000 Oe.

### Surface modification of Ni NPs

The as-synthesized hydrophobic Ni NPs were then modified to hydrophilic by water-in-oil reverse microemulsion salinization^26^. Briefly: 2 mg Ni NPs were added to 11 ml cyclohexane with 1.36 g Igepal CO-520 dissolved in the solution; the mixture was sonicated for 10 min; then 100 μl NH_3_H_2_O was added to the solution, followed by the slow addition of 10 μl TEOS; the reaction lasted for 1 h under sonication bath at the power of 450 W; the product was collected and purified by centrifugation (11,000 rpm, 5 min). The negatively charged hydrophilic Ni clusters were then modified to positively charge with PEI. Briefly: 1 ml Ni@SiO_2_ clusters (100 μg ml^−1^) were mixed with 1 ml PEI (10 mg ml^−1^); the mixture was adjusted to neutral by the addition of 122 μl HCl (1 M); then it was sonicated for 1 h at the power of 450 W; after the reaction, the particles were purified with DI water by three cycles of centrifugation (11,000 rpm, 5 min); finally, the PEI-modified Ni clusters were re-dispersed in H_2_O. Morphologies of the as-modified Ni NPs were characterized by TEM. The size and zeta potential were measured by DLS and electrophoretic light scattering (ELS), respectively.

### Synthesis of ND@pNIPAM–Ni hybrid sensor

The hybrid sensors were synthesized by electrostatic interaction of the negatively charged ND@pNIPAM particles and the positively charged Ni@SiO_2_-PEI clusters. Firstly, 1 ml ND@pNIPAM (20 μg ml^−1^ in H_2_O) and 1 ml Ni@SiO_2_-PEI (20 μg ml^−1^ in H_2_O) were mixed. Then the mixture was vibrated at 400 rpm for 30 min. Morphologies of the hybrid sensors were characterized by TEM to confirm the bonding of Ni clusters to ND@pNIPAM particles. The staining process was the same as that of ND@pNIPAM particles.

### Swelling–collapse tests of ND@pNIPAM and ND@pNIPAM–Ni

The swelling–collapse behaviors of ND@pNIPAM and ND@pNIPAM–Ni were tested by DLS with a sample concentration of 10 μg ml^−1^ in H_2_O. The hydrodynamic diameters of them at different temperatures from 25 to 50 °C were measured. The temperature of the samples was altered by the thermostatic chamber of the DLS instrument, and the uncertainty of temperature was 0.1 °C. Three cycles were conducted for each measurement. The reversibility of the hybrid sensor was tested by performing heating–cooling cycles at low temperature (25 °C) and high temperature (45 °C). Eight cycles were successively conducted at first, and then two more cycles were performed 4 days later. The temperature response (from 29.8 to 45.6 °C) of individual ND@pNIPAM particles on a Si substrate was also tested by AFM. In a typical procedure, 20 µl 10 µg ml^−1^ sample solution was dropped on a silicon wafer, which was pre-mounted on a petri dish. 2 h were allowed for the settling down of particles, then the dish was filled with DI water. Considering the softness of the pNIPAM hydrogel, a PFQNM-LC-A-CAL tip with spring constant of 0.096 N m^−1^ and a tip radius of 70 nm was used for the AFM measurement. The topography of the sample was collected with a force of 200 pN under the peak-force mode. Temperature of the solution was controlled by the ceramic heater of the system, and measured by a thermometer attached on the surface of the Si wafer. The uncertainty of temperature was 0.2 °C.

### Characterization techniques

General morphologies of the NPs were characterized using TEM (Philips CM120). All of the hydrodynamic diameters and *ζ*-potentials were measured by DLS/ELS (Malvern ZS90). AFM height profiles were acquired by Bruker BioScope Resolve. Fluorescence overlapping analysis was performed on a laser confocal microscope (Nikon Super-resolution Microscope, N-Sim). The crystallinity and phase of Ni NPs were examined by XRD (SmartLab, Rigaku) with a Cu-Kα radiation source (*λ* = 0.1541 nm). Hysteresis curves of Ni NPs were obtained by VSM (Quantum Design PPMS).

### Setup for ODMR measurement

ODMR measurements were carried out on a home-built laser scanning confocal image system (Supplementary Fig. 11). Enameled copper wires (~30 µm diameter) were used to deliver microwave to the NDs in solution. Fast frequency sweep (list-mode of SMIQ03B, 2 ms dwell time for each frequency) under multi-run was employed to average out the fluctuation of fluorescence counts, which can be caused by the laser power fluctuation and the sample drift. The sample temperature was controlled by a TEC heater and monitored by a nearby resistance thermometer. Typical heating speed was about 5 °C per min, and another 10 min were included to stabilize the solution temperature at its equilibrium state, which was confirmed by the resonant frequency of nearby NDs. Details of the temperature calibration method and data can be found in Supplementary Note 5.

### Sample preparation for ODMR measurement

The sample for the ODMR measurement was loaded by the following process. Firstly, a solution of ND@pNIPAM–Ni NPs (10 µg ml^−1^) in 50 µl H_2_O was dropped to a silicon wafer by pipette. Then the wafer was fixed on the home-built sample chamber on the printed circuit board (PCB). The sample chamber was filled with DI water (or FBS) after the settling down of the hybrid sensors (for about 20 min). Finally, the chamber was covered by a cover glass, with a small gap left to refill DI water (or FBS) for long time measurement. Details of the sample loading are given in Supplementary Note 3.

### Numerical simulation

We assumed that the normalized ODMR spectra were the average of the electron spin resonance spectra of the ensemble of NV centers inside the ND with a Lorentzian shape as^27^

1 $$S\left( f \right) = \frac{1}{N}\mathop {\sum }\limits_j \left[ {1 - C\frac{{{\mathrm{\Delta }}f^2}}{{4\left( {f - f_j^ + } \right)^2 + {\mathrm{\Delta }}f^2}} - C\frac{{{\mathrm{\Delta }}f^2}}{{4\left( {f - f_j^ - } \right)^2 + {\mathrm{\Delta }}f^2}}} \right]$$

where *N* is the number of NV centers, *C* is the contrast, Δ*f* is the FWHM, and $f_j^ \pm \approx D \pm \sqrt {E_j^2 + \gamma _e^2B_j^2}$ are the transition frequencies of the *j*th NV center spin. *E*_*j*_ is the local strain perpendicular to the axis of the *j*th NV center. The distribution of the local strains among NV centers was assumed to be a 2D Gaussian function with variance *σ*_*E*_. Typical values of Δ*f* = 9 ± 4 MHz (standard deviation of six NDs) and *σ*_*E*_ = 3.6 ± 0.5 MHz (standard deviation of six NDs) were obtained from the ODMR spectra of the bare NDs. The transition frequencies were determined by the magnetic field *B*_*j*_ projected along the axis of the *j*th NV center. The frequency shift and the broadening of the ODMR spectra correspond approximately to the mean and variance of the magnetic field, respectively. For details, see Supplementary Note 9.

### Sensitivity estimation

The shot-noise-limited sensitivity of the hybrid sensor was estimated (both in the experiments and simulations) to be^3,28^

2 $$\eta _P \approx \frac{1}{{\sqrt L }}\left| {\frac{{{\mathrm{d}}S\left( f \right)}}{{{\mathrm{d}}P}}} \right|_{{\mathrm{max}}.}^{ - 1}$$

where *L* is the photon count rate and *P* is the stimulus parameter (temperature in this paper). For details, see Supplementary Note 7.

### Data availability

The data which supports the findings of this work is available upon request from the corresponding authors.

## Electronic supplementary material

Supplementary Information
