# Supplementary material for: Hybrid nanodiamond quantum sensors enabled by volume phase transitions of hydrogels
Source: Nat Commun. 2018 Aug 9;9:3188. doi: 10.1038/s41467-018-05673-9 (PMC6085381; doi:10.1038/s41467-018-05673-9)
Supplement: Supplementary file 1 — Supplementary Information [file 41467_2018_5673_MOESM1_ESM.pdf]

Supplementary Information

for

Hybrid nanodiamond quantum sensors enabled by volume phase transitions of hydrogels

Zhang et al.

Supplementary Note 1. Numerical simulation of the magnetic field for possible configurations of the MNP cluster

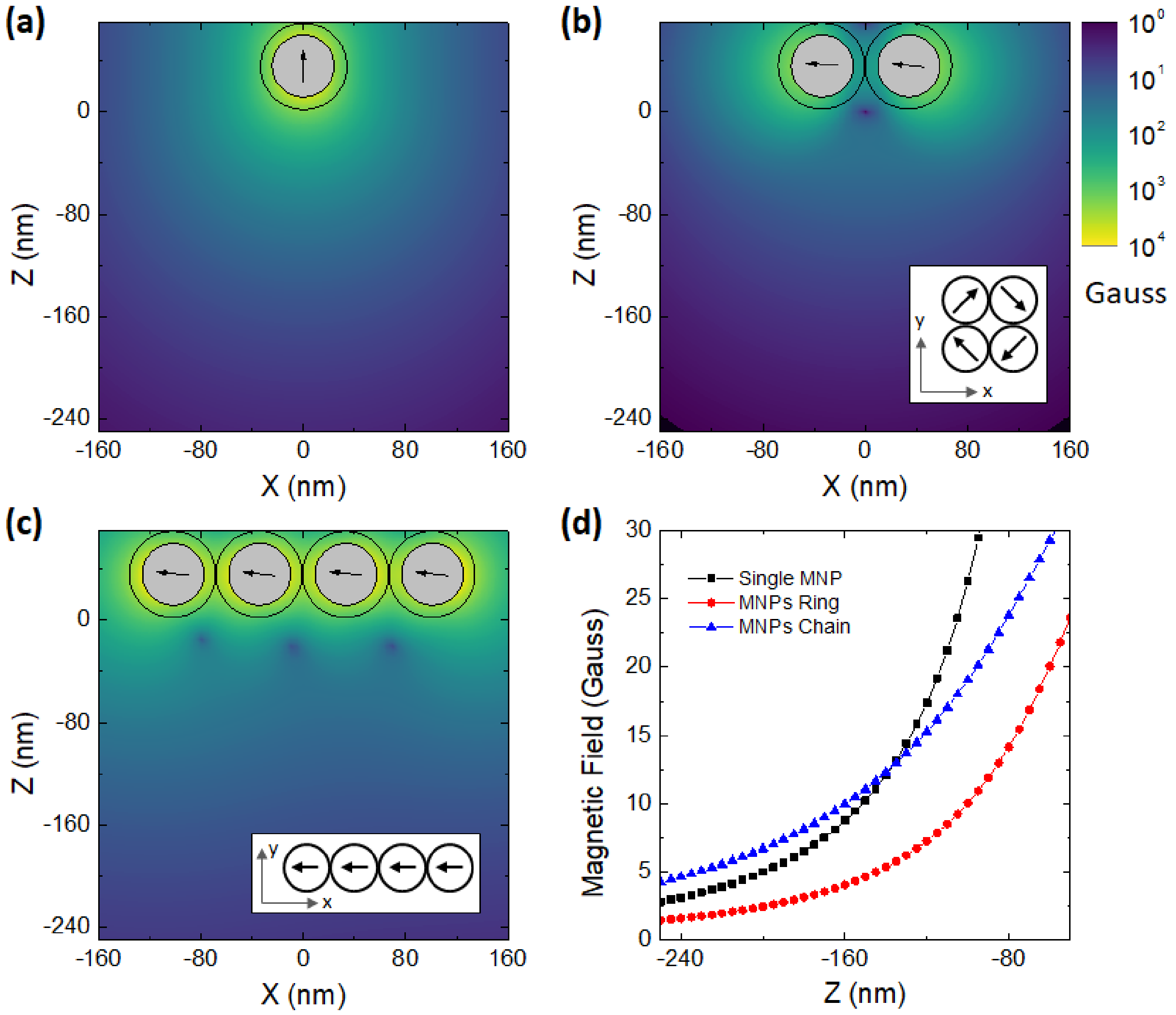

Supplementary Fig. 1: Magnetic field distributions of MNPs. Magnitude of the magnetic field from (a) a spherical Ni MNP (grey circle) with diameter 50 nm, (b) a ring cluster of 4 Ni MNPs, and (c) a chain cluster of 4 Ni MNPs. The magnetizations (indicated by the black arrows) are oriented along the ring or the chain[1] and shifted ≈ 8° out of the x-y plane. (d) The magnetic field along the z-direction at (0, 0, z) as functions of z. By comparing the magnetic fields from the cluster and the single MNP (with magnetic moment M_0, black), the effective moment of the ring (red) or the chain (blue) cluster is estimated to be ≈ 0.4 M_0 or ≈ M_0, respectively.

Supplementary Note 2. Synthesis and characterization of the hybrid sensor

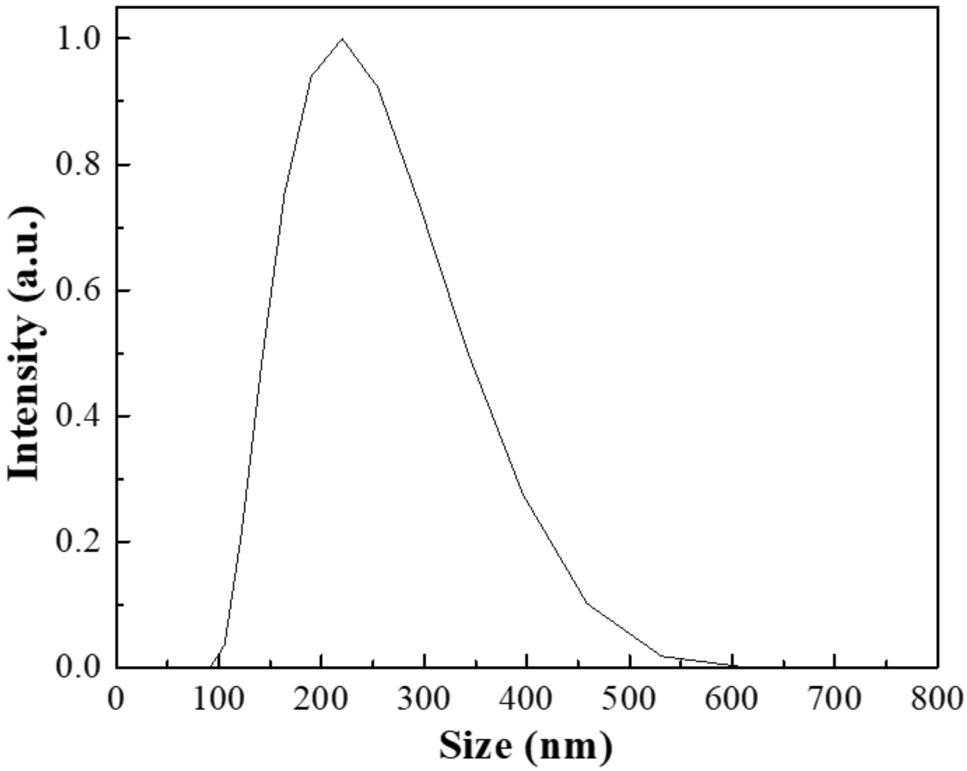

Supplementary Fig. 2: Size distribution of NDs after SiO_2 coating (ND@SiO_2), measured by DLS.

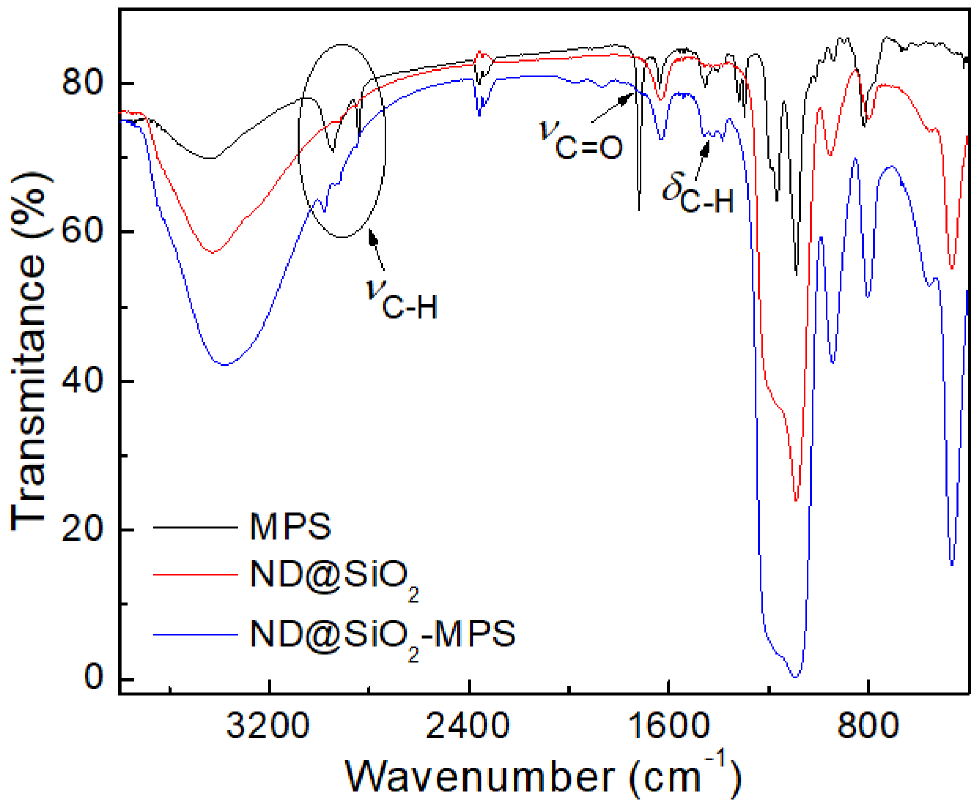

Supplementary Fig. 3: FTIR of ND@SiO$_2$ before (red) and after (blue) MPS modification, in comparison that of pure MPS (black). Peaks of ND@SiO$_2$-MPS at 2980/2850 cm^{-1} (ν(C-H)), 1720 cm^{-1} (ν(C=O)), and 1430 cm^{-1} (δ(C-H)) suggest the presence of MPS, and thus successful surface modification of ND@SiO$_2$ with MPS.

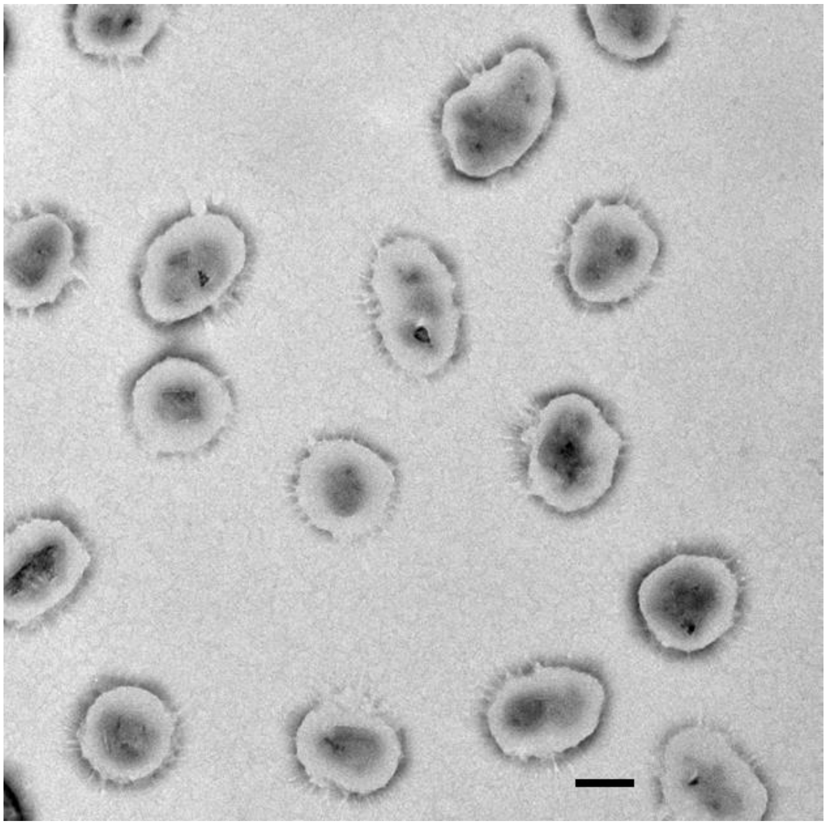

Supplementary Fig. 4: TEM image of ND@pNIPAM particles. Scale bar is 200 nm. ND@pNIPAM particles show a core-shell contrast, suggesting the successful grafting of the pNIPAM shell. The irregular ND@SiO_2 core is of darker contrast due to its larger density. The thickness of the pNIPAM shell is about 100-200 nm (in a dried state on the carbon film). The core-shell structured ND@pNIPAM particles are well dispersed on the carbon film, suggesting its excellent dispersity in H_2O at room temperature.

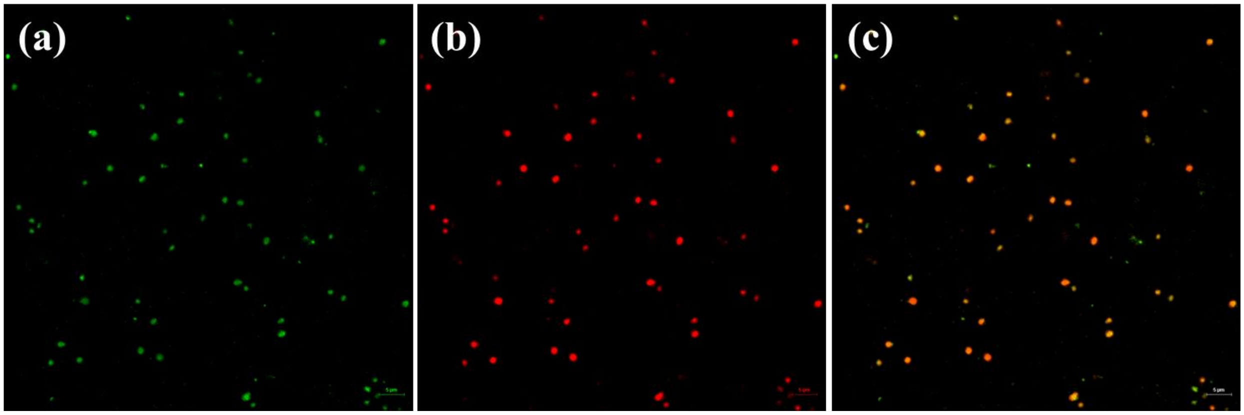

Supplementary Fig. 5: Fluorescence measurement of ND@pNIPAM. Fluorescence signal of (a) pNIPAM after FSS staining, (b) NDs, and (c) their overlay measured by N-sim. The high ratio overlap between the signals of pNIPAM and NDs suggest the effective coupling of the ND cores and the pNIPAM shells. Scale bars are 5 μm.

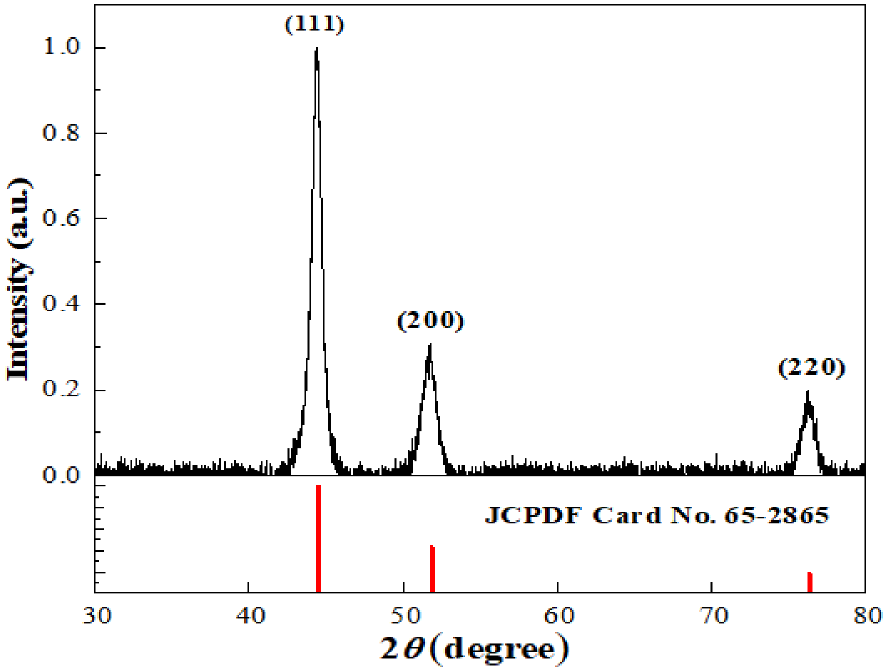

Supplementary Fig. 6: XRD spectrum of Ni NPs. The peaks of the spectrum are compared to the standard spectrum of FCC-structured Ni NPs (JCPDF Card No. 65-2865). The good match between them suggests that the as-synthesized Ni NPs are FCC-structured.

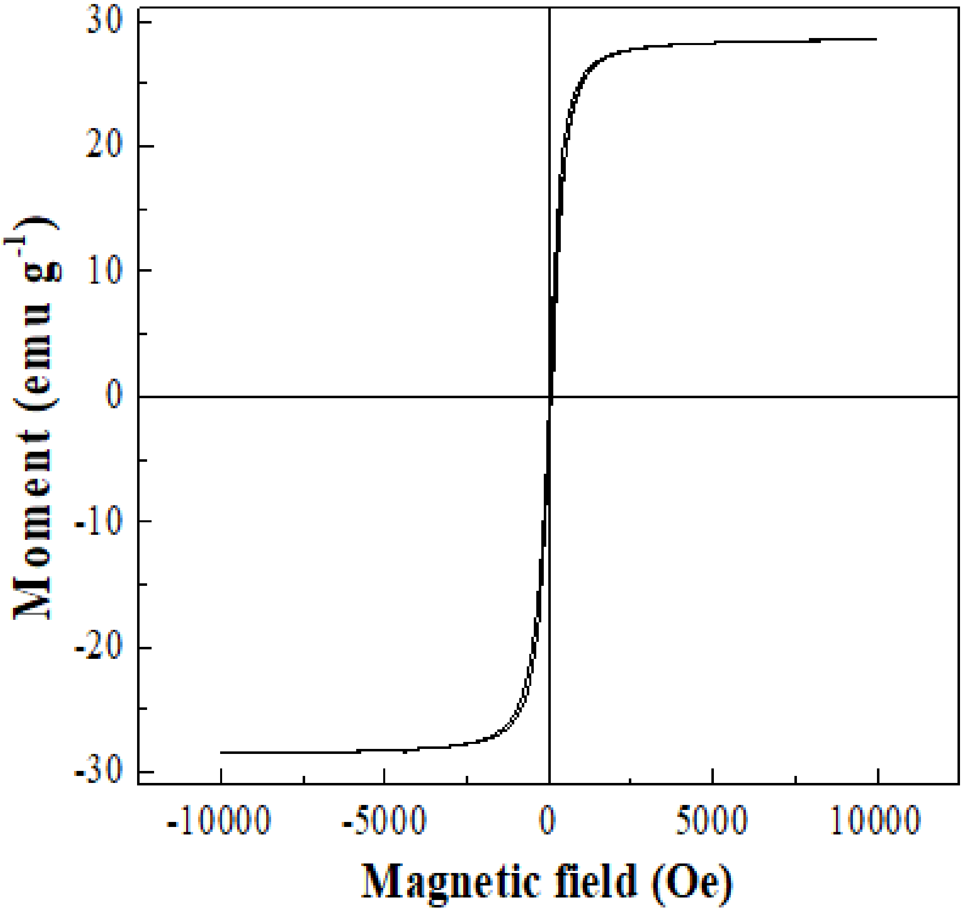

Supplementary Fig. 7: Hysteresis curve of Ni NPs measured by VSM. The saturation magnetic moment of the particles is about 28 emu g^{-1}, which is comparable with that of bulk Ni. Due to the residual magnetization of the electromagnet in VSM, together with the soft magnetism of Ni material, the coercivity and residual magnetization of the as-synthesized Ni NPs obtained from this measurement are of low accuracy.

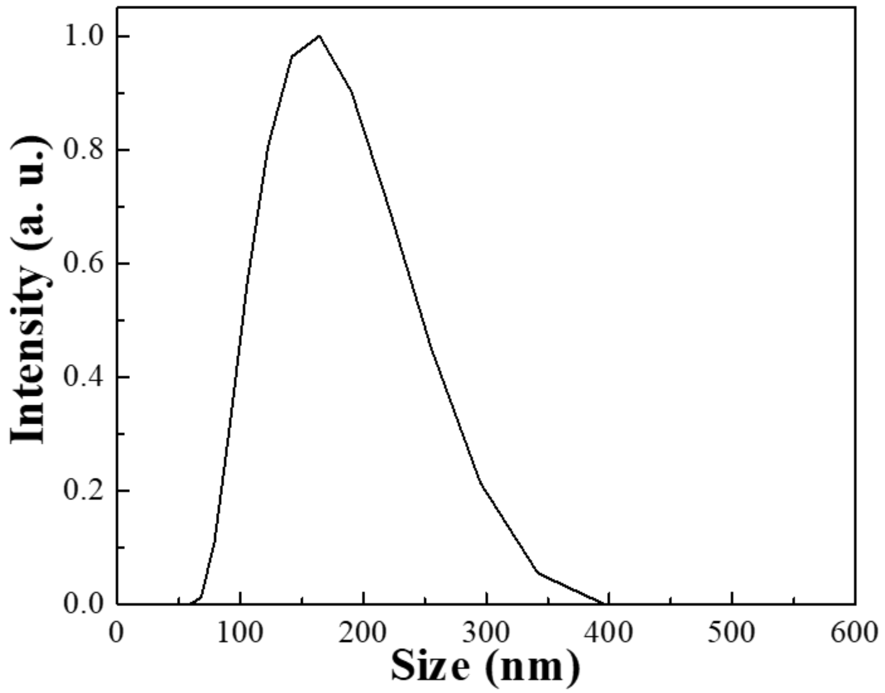

Supplementary Fig. 8: Size distribution for Ni clusters after SiO_2 coating and PEI modification (Ni-PEI).

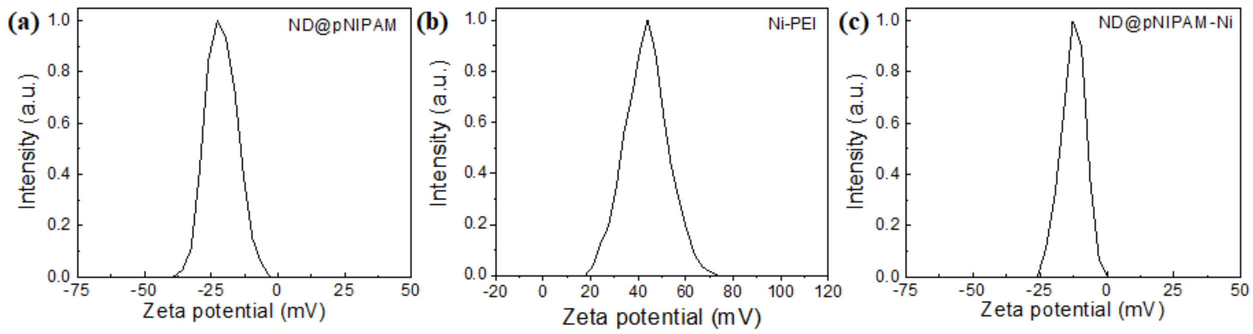

Supplementary Fig. 9: Zeta potential measurements. Zeta potential for (a) ND@pNIPAM, (b) Ni-PEI, (c) ND@pNIPAM-Ni hybrid particle.

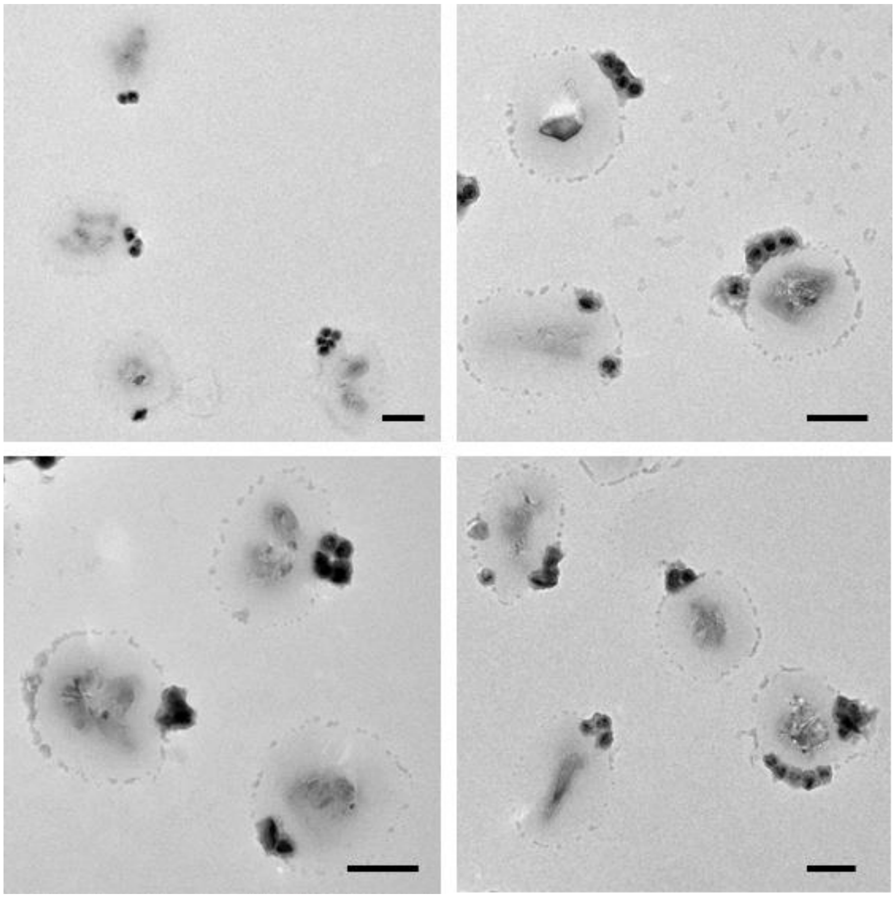

Supplementary Fig. 10: Typical TEM images of ND@pNIPAM-Ni hybrid sensors. Scale bars are 200 nm.

Supplementary Note 3. ODMR setup for the temperature sensing

A home-built sample chamber is used to carry out the temperature-dependent ODMR measurements. As shown in Supplementary Fig. 11, the body of the chamber is a printed circuit board (PCB) round hole with diameter of 20 mm and thickness of about 2 mm. The bottom of the chamber is a TEC and the top of the chamber is covered with a coverglass. Green laser beam (532 nm) is steered by a 2-axis galvo mirro system before focusing on the NDs with a 60× air objective, and the fluorescence from the NDs is collected by the same objective. Microwave (MW) pulses are delivered to the sample with enameled copper wire (30 μm diameter). A drop of water

solution (50 μl) that contains hybrid sensors is firstly transferred to the silicon substrate by pipette. And the silicon substrate is fixed on the bottom of the sample chamber. After about 20 minutes, some of the hybrid sensors settle down to the surface of the silicon substrate, then the chamber is filled with deionized water (or FBS) and covered with cover glass. The hybrid sensors are randomly distributed on the silicon substrate. Under excitation power of about 100 μW, the fluorescence counts of a single ND are about 5 M per second. The temperature of the chamber is controlled by the TEC heater and monitored with a resistance thermometer.

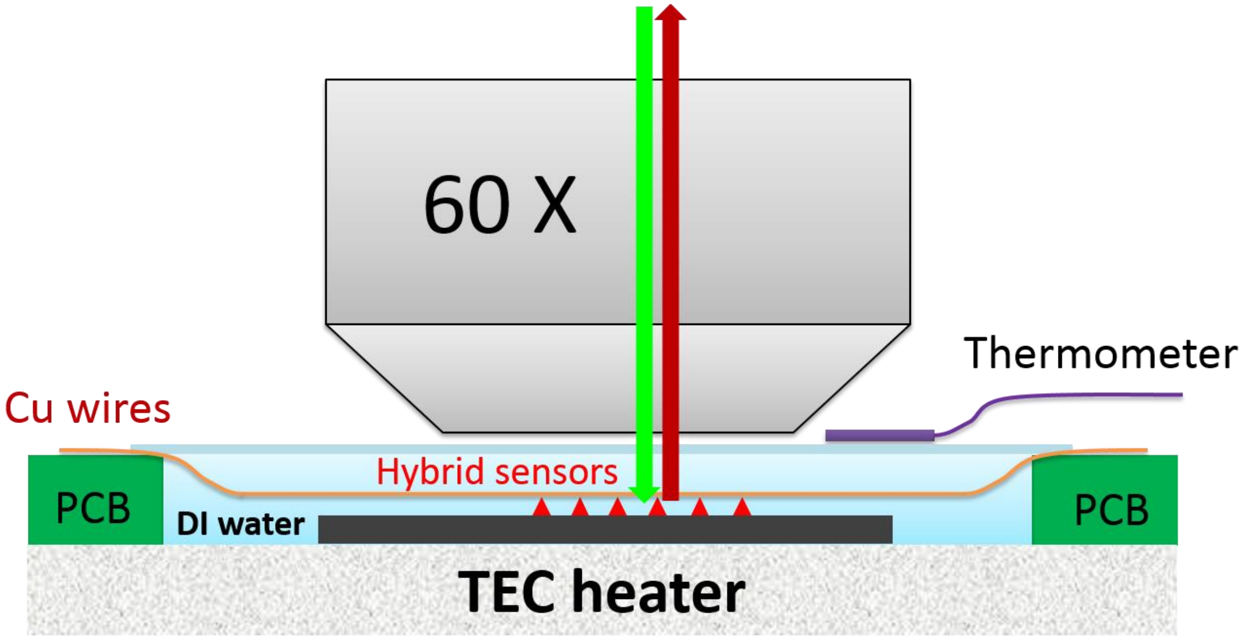

Supplementary Fig. 11: ODMR sample loading and temperature control. The home-built sample chamber is filled with deionized water (blue color in side view). The temperature of the chamber is controlled by the TEC heater and monitored by the nearby resistance thermometer (purple). Hybrid sensors (solid red triangles) are randomly distributed on the silicon substrate. Laser excitation and fluorescence collection are carried out with the same objective. MW pulses are delivered to the NDs by the copper wire (gold line).

Supplementary Note 4. Lorentzian double-peak fitting

The measured ODMR spectra $S(f)$ of both sensor NDs and bare NDs show a two-peak feature in all profiles recorded. They are normalized to the counts of off-resonance MW driving (other experiment conditions are the same). The normalized ODMR spectra are then fitted by nonlinear least-square method with the Lorentz double-peak function as,

$$\mathcal{L}(f) = 1 - C_1 \frac{\Delta f_1^2}{4(f-D-f_\mathrm{S})^2+\Delta f_1^2} - C_2 \frac{\Delta f_2^2}{4(f-D+f_\mathrm{S})^2+\Delta f_2^2}, \qquad \text{(Supplementary Equation 1)}$$

where D is the zero-field splitting of the NV centers, f_s is the peak shifts from D, $C_{1,2}$ are the contrast, and $\Delta f_{1,2}$ are the FWHM (full width at half maxinum) of the correponding Lorentz peaks. The zero-field splitting *D*, peak shifts, and FWHM of the corresponding Lorentz peaks in all plots are obtained from the fitted experimental data (for both bare NDs and ND@pNIPAM-Ni hybrid sensors).

Supplementary Note 5. Temperature calibration

Due to heat dissipation, the sample temperature is slightly higher than that measured by the resistance thermometer. We use NDs (and hybrid sensors) themselves to calibrate the local temperatures. Three assumptions are made in the temperature calibration: (1) At room temperature, when the TEC heater is turned off, the sample (NDs or hybrid sensors) and the thermometer measure the same temperature, that is, the room temperature; (2) The zero-field splitting *D* of NV centers in the ND is linearly dependent on its local temperature, with a slope of ~74 kHz K^{-1}, which is supported by literature as well as our measurements on different NDs[2]; and (3) The sample temperature has linear dependence on the TEC heater voltage.

The ODMR spectra of bare NDs are recorded at different TEC voltages, for both heating and cooling processes, as shown in Supplementary Fig. 12b, c. At each voltage, a double-peak Lorentz fitting is used to extract the resonant frequencies of the two peaks. Then the zero-field splitting D is obtained by averaging the two frequencies. Supplementary Figure 12d plots the zero-field splitting as a function of the TEC voltage, and a linear dependence can be observed. The bare ND shows a good reversibility of temperature response in the heating and cooling processes. The measurements on reference ND suggest the linear dependence of temperature on TEC voltage, as well as the consistent temperature dependence for heating and cooling processes.

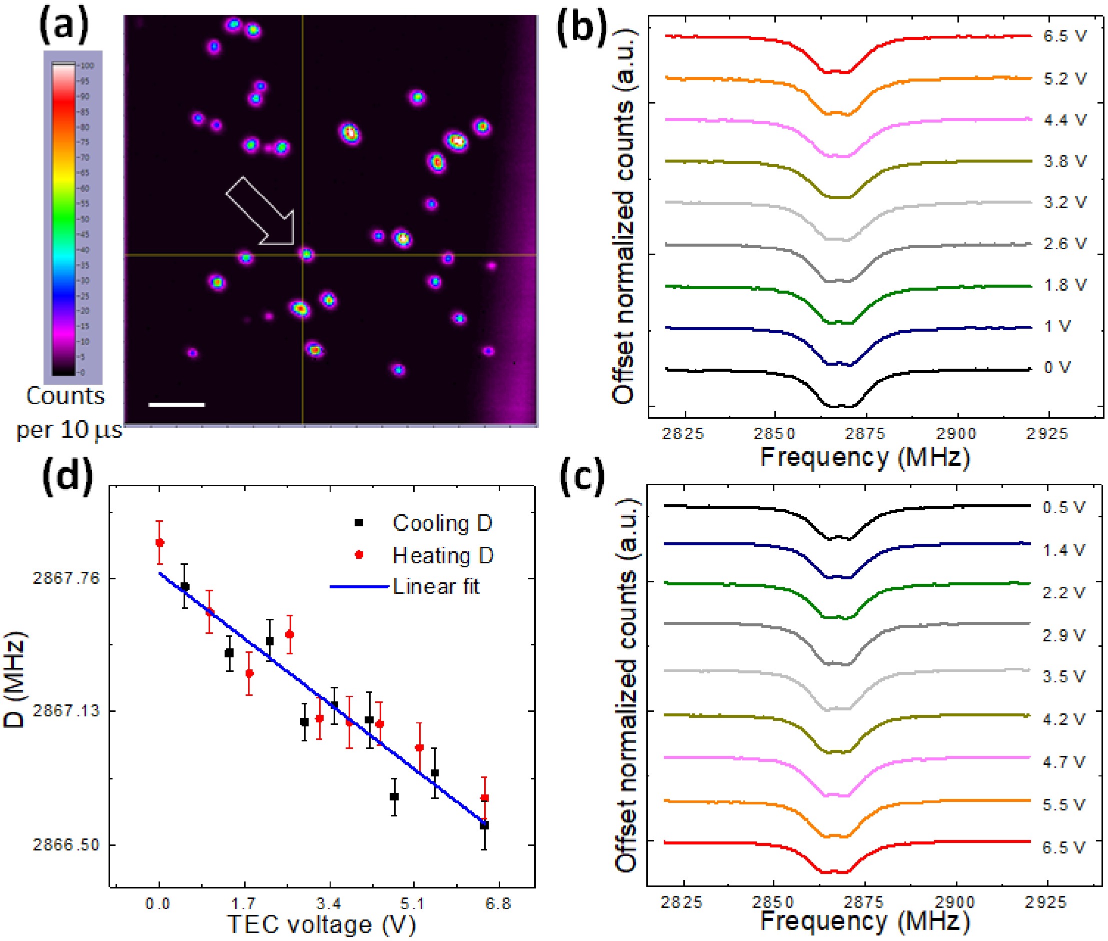

Supplementary Fig. 12: ODMR measurements of reference ND. (a) Typical confocal image of NDs on Si substrate. Scale bar is 5 µm. The marked one is a reference ND (bare ND). (b), (c)

ODMR spectra of the reference ND at different temperatures during heating and cooling processes, respectively. (d) Zero-field splitting of the reference ND (without Ni MNPs) as a function of the TEC voltage. Both heating (red) and cooling (black) processes are measured, and the blue line is linear fitting of the data. The vertical error bars of the red and black data points are the fitting errors.

Based on the assumptions and the measurements on the reference ND, the local temperature of the hybrid sensor is calibrated. Supplementary Figure 13 (left y-axis) shows the zero-field splitting extracted from the fitted spectra of the hybrid sensor (as shown in Fig. 5a, b in the main text). A linear dependence of D on the TEC heating voltage is identified as $\frac{dD}{dV} \approx 0.18 \pm 0.02\ \mathrm{MHz\ V^{-1}}$ (fitting errors). Then the dependence of temperature on TEC voltage is calculated by combining with the linear dependence of the zero-field splitting on temperature ($\frac{dD}{dT} \approx -74\ \mathrm{kHz\ K^{-1}}$). Finally, by fixing the first point as the real-time room temperature measured by the thermometer, the temperature of hybrid sensor particle is calibrated as shown in Supplementary Fig. 13 (right y-axis). In this way, a correlation between the TEC heater voltage and the real temperature at the sensor ND is established.

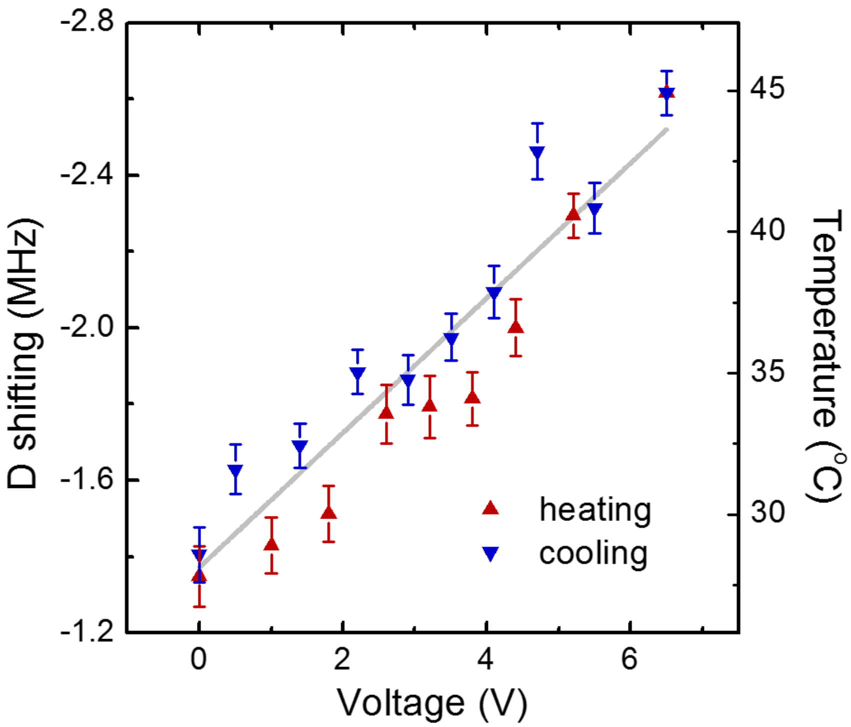

Supplementary Fig. 13: Zero-field splitting *D* and corresponding calibrated temperature as functions of the voltage of the TEC heater for the heating (red triangles) and cooling (blue triangles) processes. Grey line is the linear fitting of the zero-field splitting. The vertical error bars of the red and blue data points are the fitting errors.

Supplementary Note 6. Temperature sensing

Using the same data fitting method as stated in Supplementary Note 4, the peak shifts (Fig. 5c in main text) and the FWHM (Supplementary Fig. 14) are plotted as functions of the calibrated local temperature. Increase in both resonance frequency shifts and FWHM of the peaks are observed with temperature increase, as results of the magnetic field and field gradient increase due to collapse of the pNIPAM hydrogel.

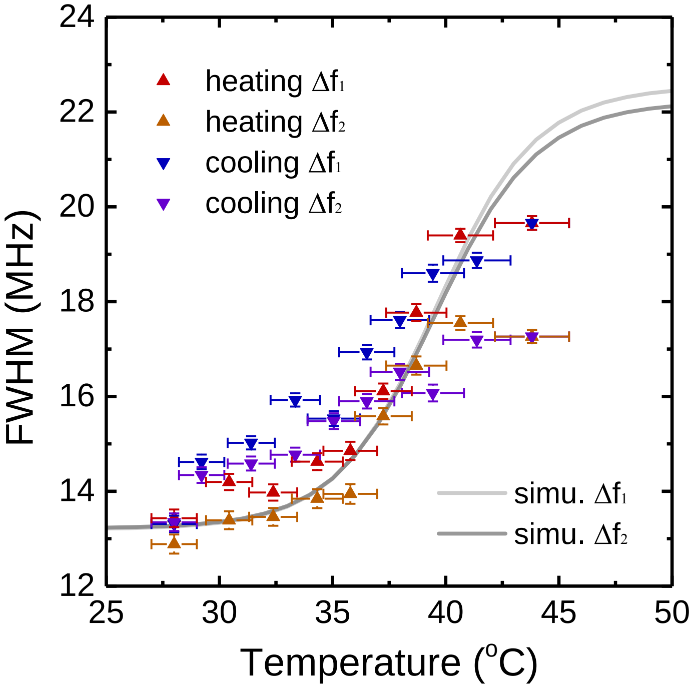

Supplementary Fig. 14: FWHMs of the two-peak fitting of the ODMR spectra as functions of the temperature for the heating (red and orange) and the cooling (blue and purple) processes, respectively. The Grey lines are the simulation result. The horizontal error bars are the fitting errors of the temperature calibrations. The vertical error bars are the fitting errors.

Supplementary Note 7. Estimation of the sensitivity of the hybrid sensor

In the real-time temperature measurement, reference temperature T_{ref} is selected and a single frequency f_{m} is chosen with maximum value of $\left|\frac{\mathrm{d}S(f)}{\mathrm{d}T}\right|_{T=T_{\mathrm{ref}}}$. By applying continuous wave ODMR measurement with MW frequency f_{m}, the temperature variation is extracted from the signal δF as

$$\delta F = \frac{\delta N_{\mathrm{m}}}{N_0}; \qquad \delta T = T - T_{\mathrm{ref}} = \delta F / \frac{\mathrm{d}S(f_{\mathrm{m}})}{\mathrm{d}T}|_{T=T_{\mathrm{ref}}}, \qquad \text{(Supplementary Equation 2)}$$

where $N_\mathrm{m} = L\Delta t S(f_\mathrm{m})$ is the photon count obtained during the ODMR measurement with L denoting the photon count rate and Δt the integration time for each temperature measurement. The spectra are normalized by $N_0 = L\Delta t$, the photon count for off-resonant MW frequency. Shot noise is obtained in the photon count measurement as $\sigma_N = \sqrt{N_\mathrm{m}}$, and the shot noise on the signal is $\delta F_\mathrm{noise} = \frac{\sigma_N}{N_0} \approx \frac{1}{\sqrt{L\Delta t}}$. For the shot-noise-limited measurement, it is assumed that $\delta F_\mathrm{noise} \approx \delta F$, and the sensitivity of the hybrid sensor is[3]

$$\eta_T = \delta T\sqrt{\Delta t} \approx \frac{1}{\sqrt{L}}\left|\frac{\mathrm{d}S(f_\mathrm{m})}{\mathrm{d}T}\right|^{-1}. \qquad \text{(Supplementary Equation 3)}$$

For comparison, the sensitivity of a bare ND (in the absence of MNPs, with the other parameters kept the same as the hybrid sensor) is estimated. The optimal resonance frequency f_m for the bare ND is chosen as $f_\mathrm{m} = D(T) + f_\mathrm{s} + \Delta f_1/(2\sqrt{3})$, and now only the zero-field splitting is dependent on temperature. Therefore, the sensitivity of bare ND is estimated to be $\eta_T^b \approx \frac{4}{3\sqrt{3}}\frac{\Delta f_1}{C_1\sqrt{L}\left|\frac{\mathrm{d}D}{\mathrm{d}T}\right|}$, which is ~0.9 K $\mathrm{Hz}^{-1/2}$ (olive dash line in Fig. 5d in the main text). In this estimation, the intrinsic parameters of the ND (such as Δf_1 and C_1) are extracted from the ODMR spectrum of the hybrid sensor at lowest temperature (28 °C). At such a low temperature, the hydrogel shell is in the fully swollen state and the Ni MNPs are far away from the ND and hence have negligible effects on the ODMR spectra of the ND.

Supplementary Note 8. Temperature sensing in fetal bovine serum (FBS)

Supplementary Figure 15a shows the ODMR spectra of one ND@pNIPAM-Ni hybrid sensor measured in FBS at a series of temperatures (using the same methods as measurements in water). The temperature-dependent peak shift f_s and FWHM $\Delta f_{1,2}$ are plotted in Supplementary Fig. 15b.

Similar to the measurements in water, both the peak shift and peak width increase with the temperature increase. An LCST of ~35 °C is estimated from the temperature-dependent peak shift/width plots (Supplementary Fig. 15b). The LCST in FBS is slightly lower than that in water (~37 °C), due to the high concentration of salt ions (>100 mM) of FBS, which is consistent with literature[4,5]. The shot-noise-limited sensitivity η_T of the hybrid thermometer in FBS is estimated (Supplementary Fig. 15c) by adopting the same method as in water. Optimal sensitivity of the hybrid sensor in FBS is estimated to be 152 ± 2 mK $\mathrm{Hz}^{-1/2}$ (shot noise error from the ODMR spectrum) around the LCST, an improvement by ~6 times from that of a bare ND (~ 0.93 K $\mathrm{Hz}^{-1/2}$, grey dashed line in Supplementary Fig. 15c). The temperature sensitivity of the hybrid sensor in FBS is similar to that measured in water.

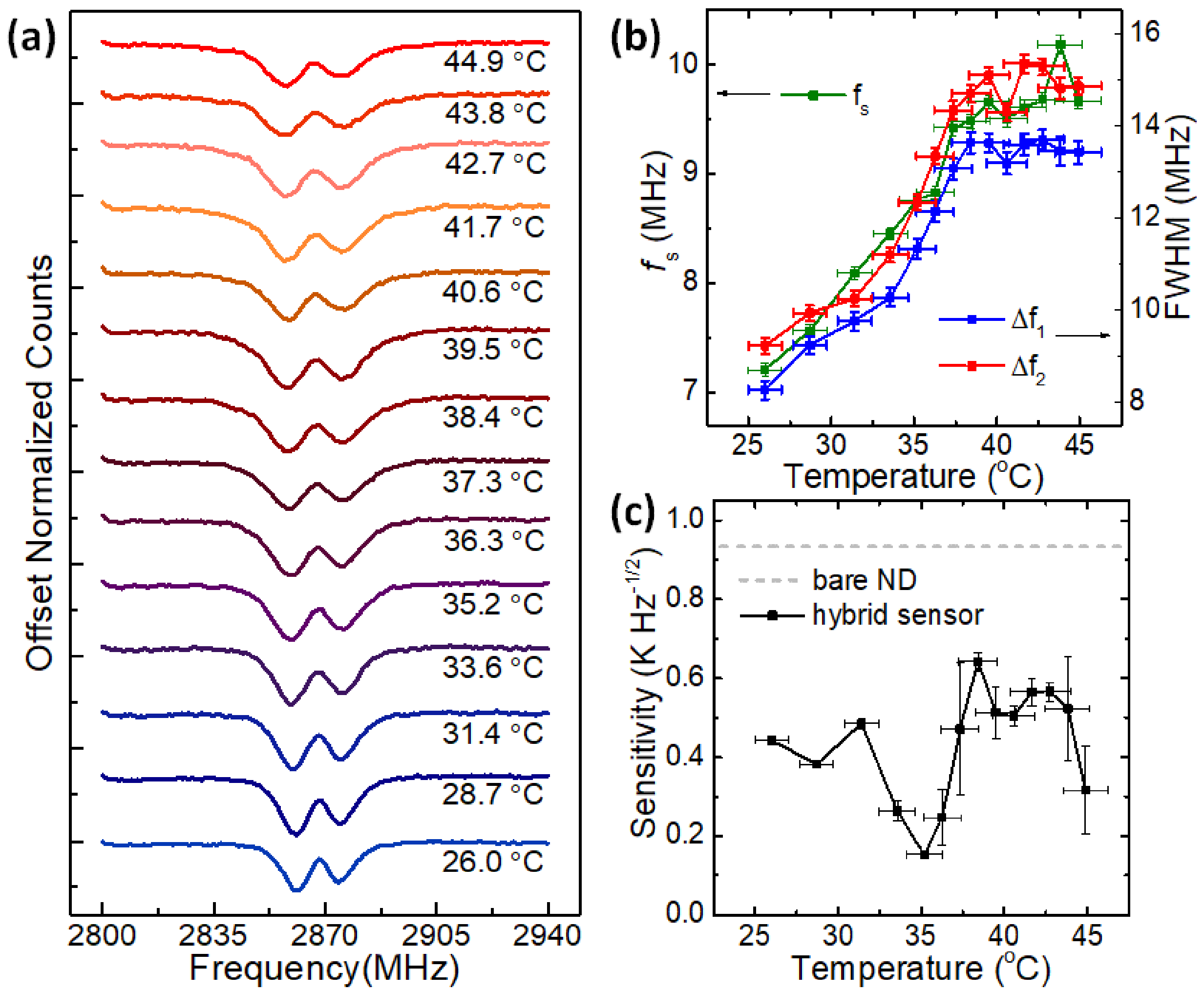

Supplementary Fig. 15 Temperature sensitivity of the hybrid sensor in FBS. (a) ODMR spectra of an ND@pNIPAM-Ni hybrid sensor at different temperatures (from top to bottom in time sequence). (b) Temperature dependence of the peak shift f_s (green) and width FWHM $\Delta f_{1,2}$ (blue and red). The vertical error bars are the fitting errors. (c) Estimated sensitivity at different temperatures (black). The grey dash line in (c) indicates the sensitivity of the bare ND temperature sensor. The vertical error bars are the shot noise errors from the ODMR spectra. The horizontal error bars in (b) and (c) are the fitting errors of the temperature calibrations.

Supplementary Note 9. Numerical Simulation of ND@pNIPAM-Ni hybrid sensor

The hybrid nano-sensor is numerically simulated with a simplified model, as illustrated in Supplementary Fig. 16a. 500 NV centers with four different orientations are uniformly distributed inside the ND, which is assumed to be a cuboid. The Ni cluster is assumed to contain four spherical Ni nanoparticles. The ND and the Ni cluster are separated by the pNIPAM with thickness h. The Hamiltonian of the NV centers becomes[6],

$$H = \sum_j H_j = \sum_j \left[D(T) S_{3,j}^2 + E_j\left(S_{1,j}^2 - S_{2,j}^2\right) - \gamma \mathbf{S}_j \cdot \mathbf{B}_j(h)\right], \quad \text{(Supplementary Equation 4)}$$

where $\mathbf{S}_j = \left(S_{1,j}, S_{2,j}, S_{3,j}\right)^{\mathrm{T}} = \mathbf{R}(\mathbf{n}_j)(S_X, S_Y, S_Z)^{\mathrm{T}}$ are the effective spins of the NV centers, $\gamma = 2.8\ \mathrm{MHz\ G^{-1}}$ is the electron gyromagnetic ratio, $D(T)$ is the zero-field splitting of the NV centers and E_j ($E_j = \sqrt{E_{X,j}^2 + E_{Y,j}^2}$) are the splitting due to local strain perpendicular to directions of the NV centers. The distribution of the local strains among NV centers is assumed to be a 2D-Gaussian function as $P(E_X, E_Y) \sim \exp\left(-\frac{E_X^2}{2\sigma_E^2}\right)\exp\left(-\frac{E_Y^2}{2\sigma_E^2}\right)$, where σ_E is the variance. $\mathbf{B}_j(h)$ is the applied magnetic field on the jth NV centers induced by the Ni MNPs. The MNPs are treated as magnetic dipoles so that

$$\mathbf{B}_j(h) = \frac{\mu_0 V}{4\pi} \sum_\nu \frac{1}{\left|\mathbf{r}_{j\nu}(h)\right|^3}\left[\mathbb{I} - \frac{3\mathbf{r}_{j\nu}(h)\mathbf{r}_{j\nu}(h)}{\left|\mathbf{r}_{j\nu}(h)\right|^2}\right]\mathbf{M}_\nu, \quad \text{(Supplementary Equation 5)}$$

where V is the volume of the Ni nanoparticles, $\mathbf{M}_\nu$ is the magnetization of the νth nanoparticle and $\mathbf{r}_{j\nu}$ is the distance between the jth NV center and the νth Ni particle, which depends on the pNIPAM thickness $h(T)$ and the temperature. Transition frequencies $f_j^{\pm}$ of the NV centers between $|\pm 1\rangle$ and $|0\rangle$ spin states then are obtained by diagonalization of the Hamiltonian H_j. Therefore, the ODMR spectra is written in summation of Lorentzian functions as

$$S(f) = \frac{1}{N}\sum_j\left[1 - C\frac{\Delta f^2}{4\left(f - f_j^+\right)^2 + \Delta f^2} - C\frac{\Delta f^2}{4\left(f - f_j^-\right)^2 + \Delta f^2}\right], \quad \text{(Supplementary Equation 6)}$$

where C is the ODMR contrast and Δf is the linewidth (FWHM) of each NV center. Typical values of $\Delta f = 9 \pm 4$ MHz and $\sigma_E = 3.6 \pm 0.5$ MHz are obtained by fitting the ODMR spectra of six bare NDs (for example, Supplementary Fig. 12b, c).

For the hybrid nano-sensor, the parameters of the NV centers in a bare ND are assumed to be $\sigma_E = 3.6$ MHz, $C = 7.5\%$, $\Delta f = 10$ MHz and the total count rate $L = 8 \times 10^6\ s^{-1}$. The relative angle between the ND and Ni cluster is set as $\theta = 130°$ (which is not optimized but the dependence of the sensitivity on the angle is weak since the ODMR spectra is averaged over NV centers along four different crystallographic directions). The dependence of pNIPAM thickness on the temperature $h(T)$ is obtained from the DLS results in Fig. 3a in the main text. Supplementary Figure 16b shows two typical simulated ODMR spectra at high (45 °C) and low (30 °C) temperature, respectively. The simulated ODMR spectra are fitted with the two-peak function described in Supplementary Note 4 (grey lines in Supplementary Fig. 16b), and the fitting parameters f_s and $\Delta f_{1,2}$ are shown by grey lines in Fig. 5c in main text and Supplementary Fig. 14, respectively. Sensitivities estimated by the simulation results are plotted by the grey line in Fig. 5d in main text. The parameters and the sensitivity are consistent with experiment results.

Sensitivities are estimated with different moments of an effective magneic dipole (which approximates a single MNP or an MNP cluster) and different thickness of the pNIPAM shell. The Ni MNPs used in our experiment form ring or chain clusters of 2-6 MNPs, as shown in Supplementary Fig. 10. The magnetic dipole-dipole interactions among the MNPs may be the driving force of the cluster formation, and therefore the magnetization orientations of MNPs should follow the cluster geometry[1]. Supplementary Figure 1b (c) shows the magnetic field of a ring (chain) of 4 MNPs. The imbalanced cancellation of the magnetizations of the MNPs in a ring cluster results in a net magnetic field similar to that from a single MNP. In the case of chain-shape

clusters, the net field is large along the chain axis but relatively weak (comparable to that from a single MNP) at the location of the ND, which is usually on the perpendicular plane of the chain. The cluster's magnetic field at the location of the ND is thus approximated as that of a magnetic dipole on the pNIPAM surface with an effective moment (in units of M_0, the moment of a single MNP). Given the thickness of the hydrogel in the fully swollen state h_{s}, the thickness at the LCST h_{c} is determined by the volume change ratio measured by DLS, with the assumption that the volume change ratio is independent of the thickness. The thickness change per Kelvin at the LCST is (for the sake of simplicity, the ND and pNIPAM are assumed to be spherical, and the ND diameter $d = 50$ nm)

$$\frac{dh}{dT}\Big|_{h=h_{\mathrm{c}}} = (R_{\mathrm{v}} - 1)\frac{(d/2+h_{\mathrm{c}})^3-(d/2)^3}{3(d/2+h_{\mathrm{c}})^2}, \qquad \text{(Supplementary Equation 7)}$$

where the volume change ratio per Kelvin at the LCST $R_{\mathrm{v}} = V(T_+)/V(T_-) \approx 0.84$ is measured by DLS (see Fig. 3a in the main text). The optimal sensitivity versus the effective moment of the MNP cluster and the thickness of pNIPAM (h_{s}) is shown in Supplementary Fig.16c. In most cases, the optimal sensitivity is 60-200 mK $\mathrm{Hz}^{-1/2}$ for hydrogel thickness from 80 to 300 nm and effective moment from 0.1 to 2 M_0.

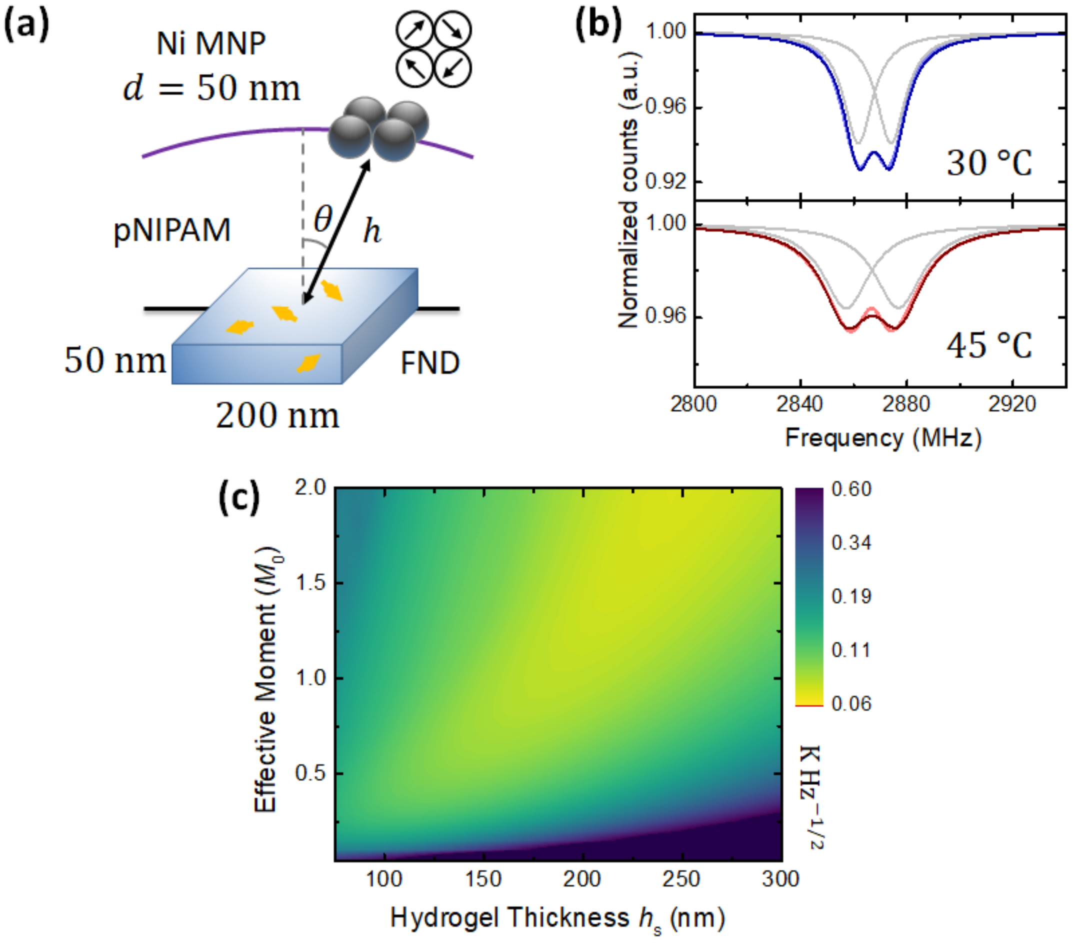

Supplementary Fig. 16 Simulation of the hybrid sensor. (a) Simplified model of the ND@pNIPAM-Ni hybrid sensor for simulation. The insert shows the orientations of magnetizations of the Ni nanoparticles. (b) Typical simulated ODMR spectra and the corresponding two-peak fitting results at high (45 °C, red, lower pane) and low (30 °C, blue, upper pane) temperatures. (c) The sensitivity at the LCST as a function of the effective moment of the MNP cluster and the fully swollen thickness of pNIPAM.

To improve the sensitivity, a hybrid sensor is constructed with pNIPAM hydrogel in-between a smaller sized ND (~50 nm in cubic shape) and a Co MNP with different diameter (Co MNPs have large magnetization at room temperature ($1{,}422\ \mathrm{kA\ m^{-1}}$) and also large single-domain size

(~ 96 nm)) [7]. Sensitivity based on the modified configuration of the hybrid sensor is simulated. The estimated sensitivity versus the diameter of the Co MNP and the thickness of pNIPAM (h_s) is mapped as shown in Supplementary Fig. 17a. The parameters of the ND are assumed to be the same as realized in the experiment (as shown in Supplementary Equation 6). An optimal sensitivity can be archived as $\sim 31\ \mathrm{mK\ Hz^{-1/2}}$ at a polymer thickness (in swollen state) of ~ 380 nm, and an MNP magnetic moment about 17 times that of a Ni MNP of 50 nm diameter, as illustrated in the main text.

Sensitivity can be further improved by using NDs that contain single NV centers. Broadening of the ODMR spectra induced by the magnetic field gradient is absent in such NDs. Simulations based on single-NV NDs are run with contrast $C = 20\%$, FWHM $\Delta f = 5$ MHz and count rate $L = 2 \times 10^5\ \mathrm{s^{-1}}$. The estimated sensitivity at the LCST versus the diameter of the Co MNP and the thickness of pNIPAM (h_s) is shown in Supplementary Fig. 17b. An optimal sensitivity $\sim 0.3\ \mathrm{mK\ Hz^{-1/2}}$ is reached when the thickness of pNIPAM in the swollen state is ~ 50 nm.

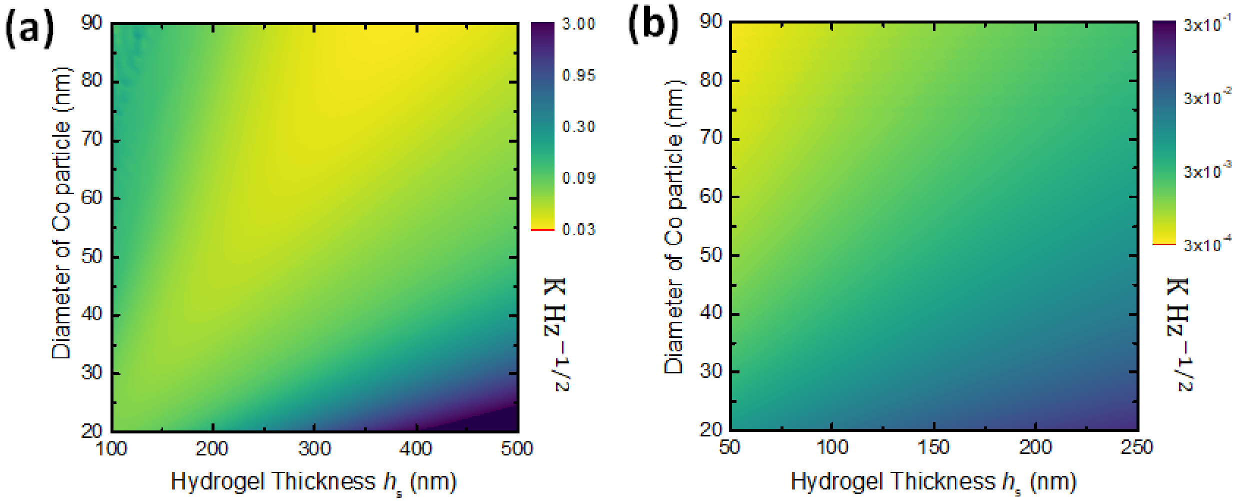

Supplementary Fig. 17 The sensitivity at the LCST as a function of the diameter of the Co MNP and the fully swollen thickness of pNIPAM. In (a) the ND contains 100 NV centers, and in (b) a single NV center. The ND has diameter of 50 nm.

Supplementary References

1 Wei, A., Kasama, T. & Dunin-Borkowski, R. E. Self-assembly and flux closure studies of magnetic nanoparticle rings. *J. Mater. Chem.* **21,** 16686-16693 (2011).

2 Acosta, V. M. et al. Temperature dependence of the nitrogen-vacancy magnetic resonance in diamond. *Phys. Rev. Lett.* **104,** 070801 (2010).

3 Taylor, J. et al. High-sensitivity diamond magnetometer with nanoscale resolution. *Nat. Phys.* **4,** 810-816 (2008).

4 Zhang, Y., Furyk, S., Bergbreiter, D. E. & Cremer, P. S. Specific ion effects on the water solubility of macromolecules: PNIPAM and the Hofmeister series. *J. Am. Chem. Soc.* **127,** 14505-14510 (2005).

5 Taha, M., Gupta, B. S., Khoiroh, I. & Lee, M.-J. Interactions of Biological Buffers with Macromolecules: The Ubiquitous "Smart" Polymer PNIPAM and the Biological Buffers MES, MOPS, and MOPSO. *Macromolecules* **44,** 8575-8589 (2011).

6 Acosta, V. et al. Temperature dependence of the nitrogen-vacancy magnetic resonance in diamond. *Phys. Rev. Lett.* **104,** 070801 (2010).

7 Guimarães, A. P. *Principles of Nanomagnetism Ch. 3* (Springer International Publishing AG, Switzerland, 2017).
